# Mapping mismanaged plastic waste in Indonesia: subdistrict-level analysis through material flow from sources to the environment

**DOI:** 10.1038/s41598-026-41849-w

**Published:** 2026-03-13

**Authors:** Attar Hikmahtiar Ramadan, Emenda Sembiring, Benno Rahardyan, Hadi Kardhana

**Affiliations:** 1 Doctoral Program of Environmental Engineering, Faculty of Civil and Environmental Engineering, Institut Teknologi Bandung, Bandung, 40132 Indonesia; 2 Air and Waste Management Research Group, Faculty of Civil and Environmental Engineering, Institut Teknologi Bandung, Bandung, 40132 Indonesia; 3 Water Resources Engineering Research Group, Faculty of Civil and Environmental Engineering, Institut Teknologi Bandung, Bandung, 40132 Indonesia

**Keywords:** Plastic pollution, Material flow analysis, Waste management, GIS, Indonesia, Environmental policy, Environmental sciences, Environmental social sciences

## Abstract

While Indonesia is a major contributor to global plastic pollution, national-level data obscures critical local hotspots where mismanagement is most severe. This study presents the comprehensive subdistrict level analysis of plastic waste flows across Indonesia to identify these hotspots and quantify their mismanagement pathways. Using a Material Flow Analysis (MFA) framework combined with Geographic Information Systems (GIS), we analyzed data from the Indonesia National Waste Management Information System (SIPSN), sampling and other governmental sources to model the fate of plastic waste. We estimate that 9.20 ± 1.52 million tonnes (Mt) of plastic waste are generated annually. This study focuses specifically on mismanaged plastic waste (MPW) that can potentially enter the environment and leak to waterways. While open burning is the largest single pathway (5.15 ± 0.68 Mt), this analysis concentrates on the 1.16 million tonnes that potentially leak into the environment. This includes 0.64 ± 0.11 Mt disposed directly into rivers and drains and 0.52 ± 0.09 Mt disposed illegally onto land. The analysis also reveals the critical role of the informal sector, which recovers four times more plastic than formal facilities (12% vs. 3%).

**Supplementary Information:**

The online version contains supplementary material available at 10.1038/s41598-026-41849-w.

## Introduction

Since the 1950 s, there has been a substantial increase in plastic production driven by the versatility and wide-ranging characteristics of plastic materials, which make them suitable for diverse applications. Despite the numerous benefits associated with plastics, such as durability and flexibility, their persistent nature and widespread usage have led to the accumulation of significant amounts of waste. This waste poses considerable challenges in terms of decomposition and disposal, contributing to environmental pollution and ecological damage. Previous studies indicate that a staggering 8,300 million tonnes (Mt) of plastic was produced globally from 1950 to 2015, with 79% of this amount ending in the open environment or landfills. If the rates of plastic production persist and the efficiency of waste management systems remain the same, projections suggest that 12,000 Mt of plastic waste will be present in the environment or landfills by 2050^1^.

Indonesia, with its large population and suboptimal waste management practices, has significant potential for substantial plastic pollution reaching the ocean. According to World Bank, in 2019, 40% of Indonesia’s 142 million urban residents still lacked access to waste collection services^2^. Suboptimal waste management leads to widespread environmental degradation, including the pollution of land and water bodies, harm to wildlife, microplastic contamination, habitat degradation, chemical pollution, and negative impacts on aesthetics and recreational activities. Moreover, this condition makes Indonesia the world’s second-largest contributor to plastic waste leakage into the sea after China based on first plastic leakage global model^3^. Further studies revealed that Indonesia is the 9^th^ largest contributor mismanaged plastic waste (MPW) globally^4^. In 2021, the study shows that Indonesia was the 5^th^ annual plastic emission into the ocean via river transport^5^. The discrepancy in Indonesia’s ranking is primarily driven by the evolution of modeling methodologies from static, population-based estimates to dynamic, hydrological frameworks. Early studies relied on a broad scope that equated mismanaged plastic waste generated within 50 km of the coast with potential leakage, often using national-level data that simplified Indonesia’s complex geography^3^. In contrast, more recent models utilize higher-resolution data and a narrower scope focused on riverine transport, accounting for variables like high tropical rainfall, terrain slope, and the probability that waste will actually reach a river mouth^5^. A recent study, specifically focused on Indonesia, estimated plastic leakage into the sea at 201,000 to 552,300 tonnes per year, with a significant portion transported through rivers^2^. In recent years, research regarding plastic leakage into the ocean has been the main topic of plastic pollution research. To complement and trace the plastic pollution that leads to marine litter, we investigated the amount of mismanaged plastic waste in Indonesia. While most previous studies tended to build models via a top-down approach based on average national data and economic classes based on GDP^2–7^, this study utilized a bottom-up methodology. This method relies on granular data available in Indonesia, incorporating normalization and uncertainty scaling to achieve a more accurate and detailed understanding on the basis of more localized sources.

Plastic leakage (PL) is broadly defined as mismanaged macro- and microplastics that ultimately enter the environment^8^. This leakage can occur at any stage of the plastic life cycle but is prominent during disposal because of illegal dumping and inadequate waste management^9^. A primary concern is that this leaked plastic becomes marine debris, which harms wildlife, carries contaminants, and threatens human health^10^. To better quantify this issue, this study divides mismanaged plastic waste on the basis of its potential for leakage. First, the potential leakage mismanaged plastic waste in this paper is categorized into two types on the basis of data available in Indonesia, namely, plastic waste disposed into the terrestrial environment (open land) and directly disposed into water bodies. Buried and openly burned plastic waste are types of mismanaged plastic waste based on previous studies^6,11–13^, however, in this study, we specified these types of mismanaged plastic waste as nonpotential leakage mismanaged plastic waste.

Currently, Indonesia’s comprehensive strategy for addressing plastic pollution encompasses a National Action Plan focusing on waste management, public awareness, stakeholder collaboration and research development, alongside measures such as a city/regency wide banning of single-used plastic bag, implementation of Extended Producer Responsibility (EPR) frameworks, investment in waste management infrastructure, support for community-based initiatives and engagement in partnerships and international collaboration. This research addresses this critical knowledge gap by conducting the first comprehensive material flow analysis (MFA) of plastic waste in Indonesia at the subdistrict (*kecamatan*) level. By integrating national waste data with high-resolution administrative boundaries and population data in a geographic information system (GIS), this study aims to (1) quantify the generation of plastic waste at a subdistrict resolution; (2) map the flows of both managed and mismanaged plastic waste; and (3) identify specific mismanagement hotspots and their dominant disposal pathways (e.g., open burning, land disposal, river disposal). The previous research has established critical baselines for marine plastic pollution these studies typically rely on top-down aggregation or localized spot sampling that obscures spatial heterogeneity^2,14^. Recent study by Cottom et al.^6^ provides a city/regency level to global inventory derived from predictive modeling using random forrest machine learning. This study advances the field by presenting the first comprehensive, bottom-up Material Flow Analysis (MFA) at the subdistrict (*kecamatan*) level for the entire Indonesian archipelago. This study also introduces a typology-based calibration framework that separately adjusts rural and urban areas to capture structural differences. By integrating granular data from the sampling, National Waste Management Information System (SIPSN)^15^ and Basic Health Research (Riskesdas 2019)^16^ with Data Quality Index (DQI) and specific calibration we explicitly disaggregate mismanaged plastic waste (MPW) into specific flows (potential leakage MPW and non-potential leakage MPW). Furthermore, the application of a multilayer Monte Carlo uncertainty framework provides the first probabilistically nationwide heatmap of land-based leakage sources. The resulting analysis provides the data necessary for evidence-based decision-making, enabling policymakers to target interventions where needed.

## Results

### Plastic waste generation and managed plastic waste

**Fig. 1:**
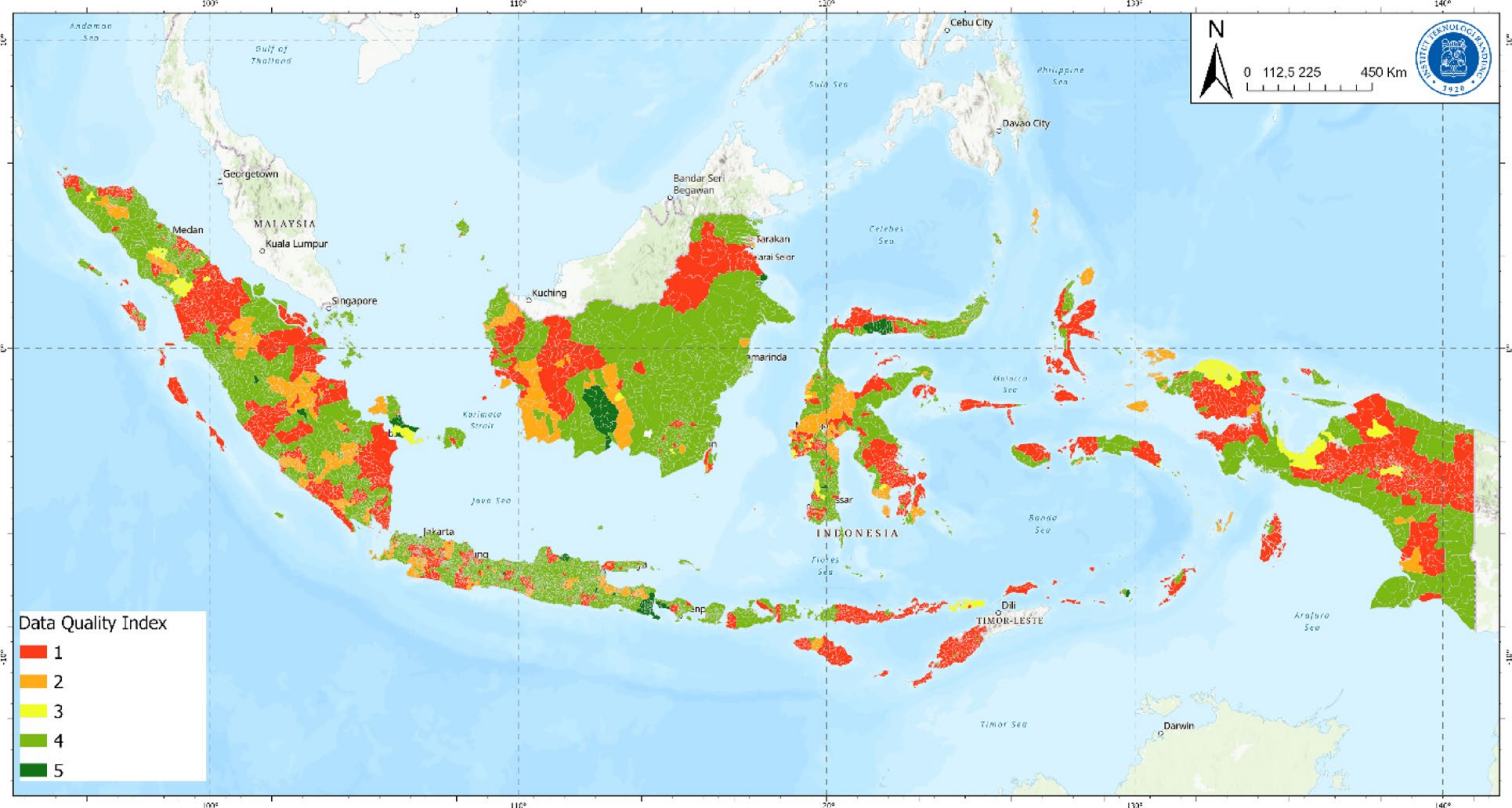
Data quality index of waste generation.

The total waste generated across all subdistricts in Indonesia is 0.14 Mt per day or 53 Mt per year, which is lower than the SIPSN (National Waste Management Information System) estimate of 70 Mt per year in 2022^15^. Moreover, plastic generation is obtained by multiplying the waste generation in each subdistrict by the percentage composition of plastic in each area. There are more empty entries in the composition data than in the available data, which is limited to the district level. For missing data, the national average plastic composition in Indonesia, which is based on SIPSN data for 2023 at 19%, is used. This study uses uncertainty analysis and data quality index (Q1-Q5). Q5 represents the highest quality data derived from direct sampling, accounting for 2% of the analysis. The majority of subdistricts, 55%, are classified as Q4, indicating recent secondary data availability. Conversely, 31% are classified as Q1, the lowest quality tier, where data gaps were filled using average values from the same province. The remaining 12% fall into medium quality (older available data) categories (Q2 and Q 3), the distribution of data quality index is shown in Fig. 1. The upper uncertainty bound (CV = 0.50) was empirically justified through validation of official SIPSN waste generation data against field measurements across 17 municipalities. The comparison yielded a mean absolute percentage error (MAPE) of 37% and an empirical reporting-error CV of 0.46. No significant systematic bias was detected (paired t-test, *p* > 0.05), indicating that dispersion rather than directional skew dominates uncertainty. The comparison data and analysis between sampling and SIPSN data is available in Suplementary Material 1. Accordingly, the 50% CV assigned to filling the unavailable data reflects realistic upper-bound volatility observed in practice. Uncertainty in both waste generation and composition was propagated through Monte Carlo simulation, and results are reported as medians with 95% confidence intervals. This probabilistic framework ensures that spatial variability in data reliability is explicitly reflected in leakage estimates. We are also introducing the calibration process refines the initial model estimates by integrating empirical sampling data specific to Indonesia’s distinct rural, semi-urban, and urban typologies. This calibration incorporates local sampling studies data to resolve the overestimation typical of broad national proxies. Consequently, the model more accurately captures the distinct waste generation profiles inherent to rural landscapes.

**Fig. 2:**
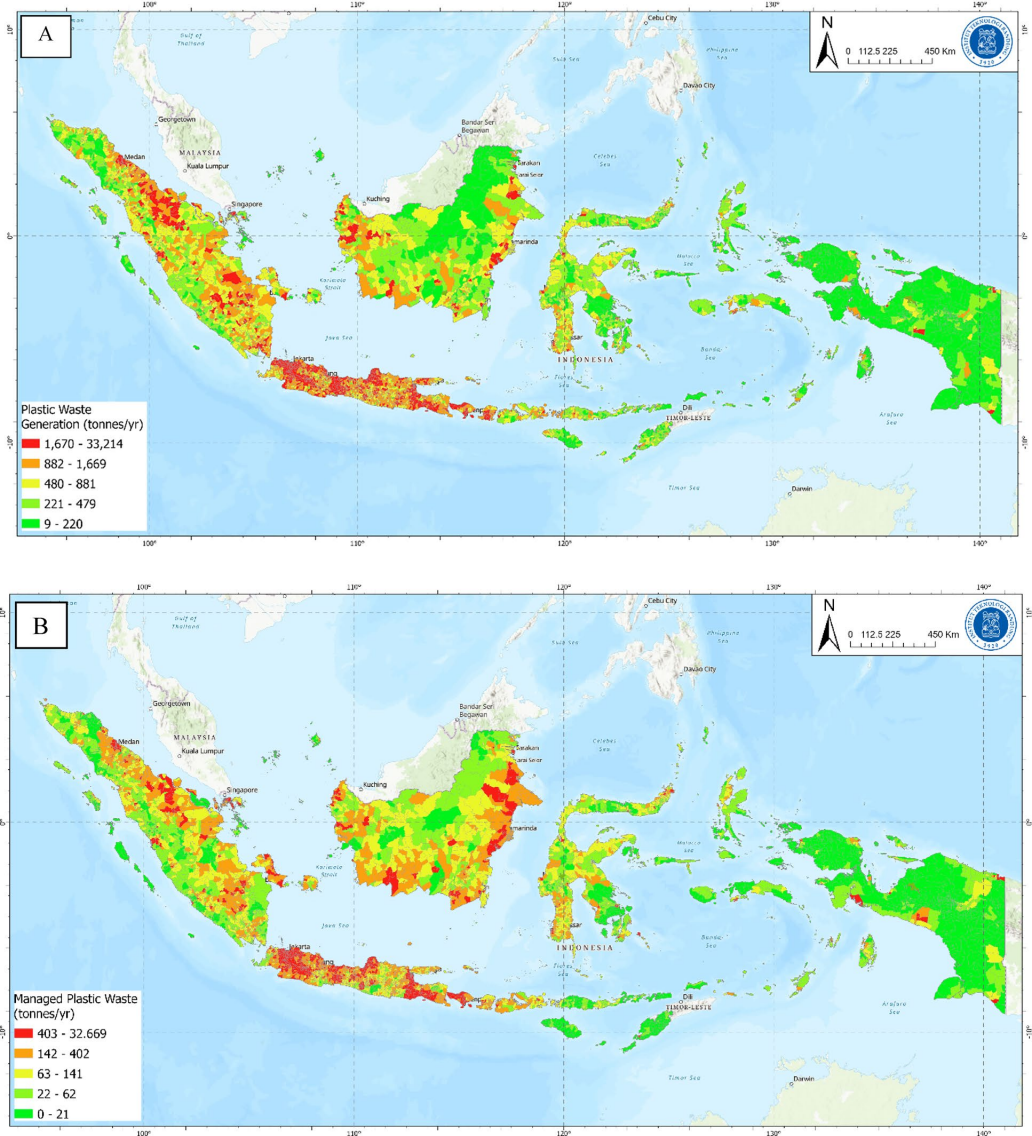
(**A**) Plastic waste generation in Indonesia, (**B**) Managed plastic waste.

An analysis of plastic waste generation in each subdistrict (kecamatan) in Indonesia revealed that plastic generation amounts approximately 25,000 tonnes per day or approximately 9.21 ± 1.52 Mt per year. Figure 2A shows the distribution of plastic waste generated at the subdistrict level. Our analysis integrates three primary data sources, namely, waste management facility records, municipal waste management performance data from the National Waste Management Information System (SIPSN), and the 2019 Basic Health Research (Riskesdas) dataset. A comprehensive list of these facilities and their respective data URLs is provided in the Supplementary Information (Table S.1)^15,16^. The analysis of plastic waste that was collected across all regions of Indonesia revealed a daily total of 10,300 tonnes or 3.77 ± 0.62 Mt annually. Moreover, the area with the largest amount of managed waste is DKI Jakarta Province, which is attributed to its largest and most densely populated districts, resulting in the highest waste generation, coupled with the highest waste management service in Indonesia. A map of managed plastic waste in Indonesia is shown in Fig. 1B.

### Potential leakage of mismanaged plastic waste

The waste handling data at the source (household) in this study are derived from the Basic Health Research published in 2019^16^, which was conducted by the National Institute of Health Research and Development to gather information on health status and determinants in Indonesia^16^. The survey includes questions regarding sanitation conditions and waste management practices at the source, with interviews conducted with hundreds of waste generators in each regency/city, averaging 432 interviews per municiaplity across Indonesia in Riskesdas (2019)^16^. These data encompass various waste treatment methods at the source, including the collection of waste as managed waste and mismanaged waste, including open burning, burying, disposal directly into the terrestrial areas (open land), and disposal into rivers or streams.

**Fig. 3:**
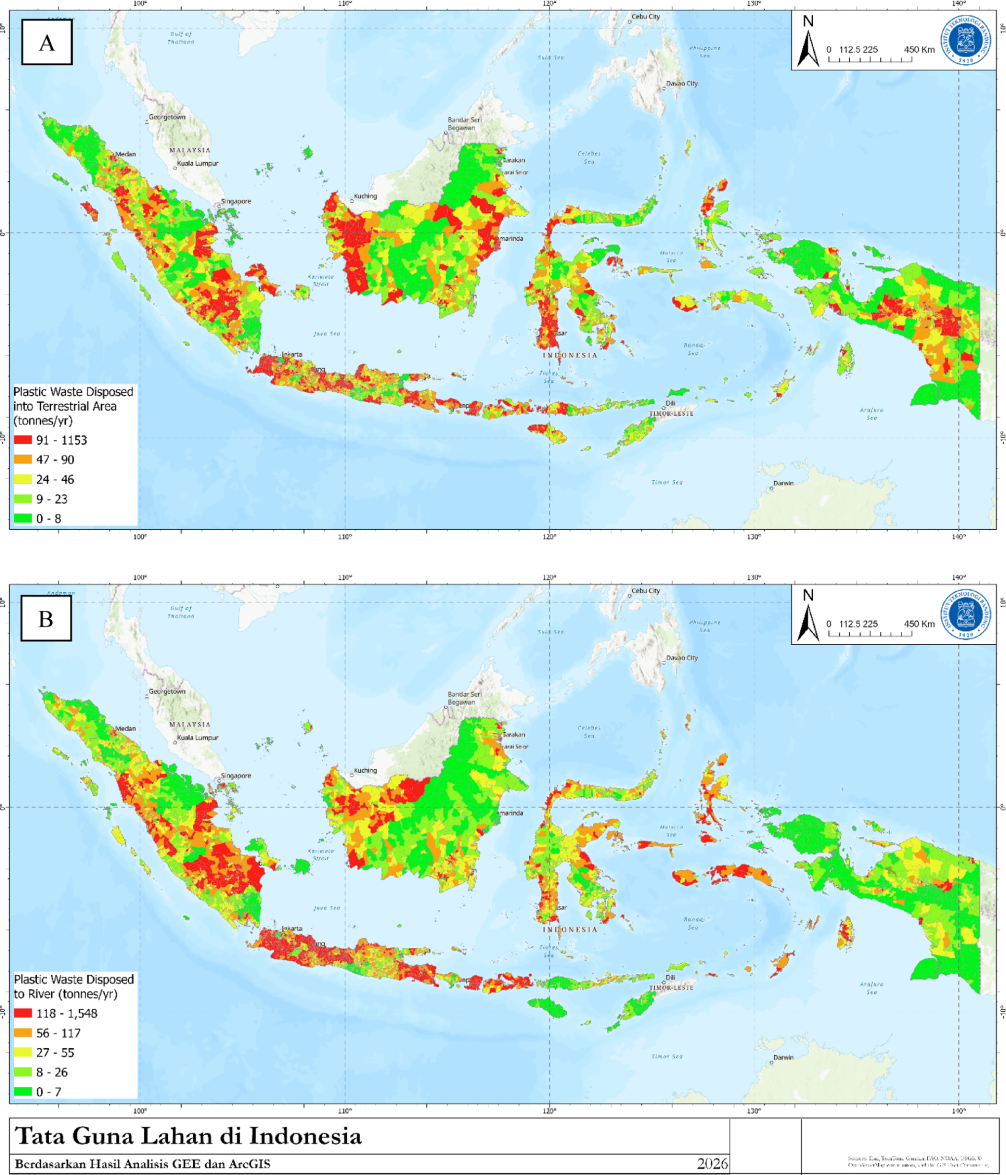
(**A**) Plastic disposed into terrestrial area, (**B**)Plastic waste disposed to river.

Based on the analysis, the direct disposal into terrestrial area (open land) of plastic waste across Indonesia amounts to 1,400 tonnes per day or approximately 0.52 ± 0.08 Mt per year (Fig. 3A). The areas with the highest rates of plastic waste mismanagement are predominantly in the subdistricts of rural regencies of Papua. High levels of mismanaged plastic waste, as evidenced in this study, are predominantly found in rural or regency areas, aligning with previous research in Indonesia indicating that rural areas contribute approximately two-thirds of mismanaged plastic waste^2^.

Rivers and drainage are among the main routes for transporting plastic from land to seas^17–19^. On the basis of the calculations conducted, the total amount of waste directly entering rivers/canals/drainage systems in the study is approximately 1700 tonnes per day or 0.64 ± 0.11 Mt per year (Fig. 3B). While Maluku and Papua have the critical highest percentages of direct disposal to rivers (13%) and direct disposal on land (15%), their absolute total waste (0.13 Mt) remains low compared to Java, meaning their overall environmental contribution is smaller despite high mismanagement rates. Several factors influence direct waste disposal into the environment, as found in a study on the Badung River in Bali^20^, including practicality, the lack of waste disposal facilities, and entrenched cultural norms.

### Non-potential leakage mismanage plastic waste

**Fig. 4:**
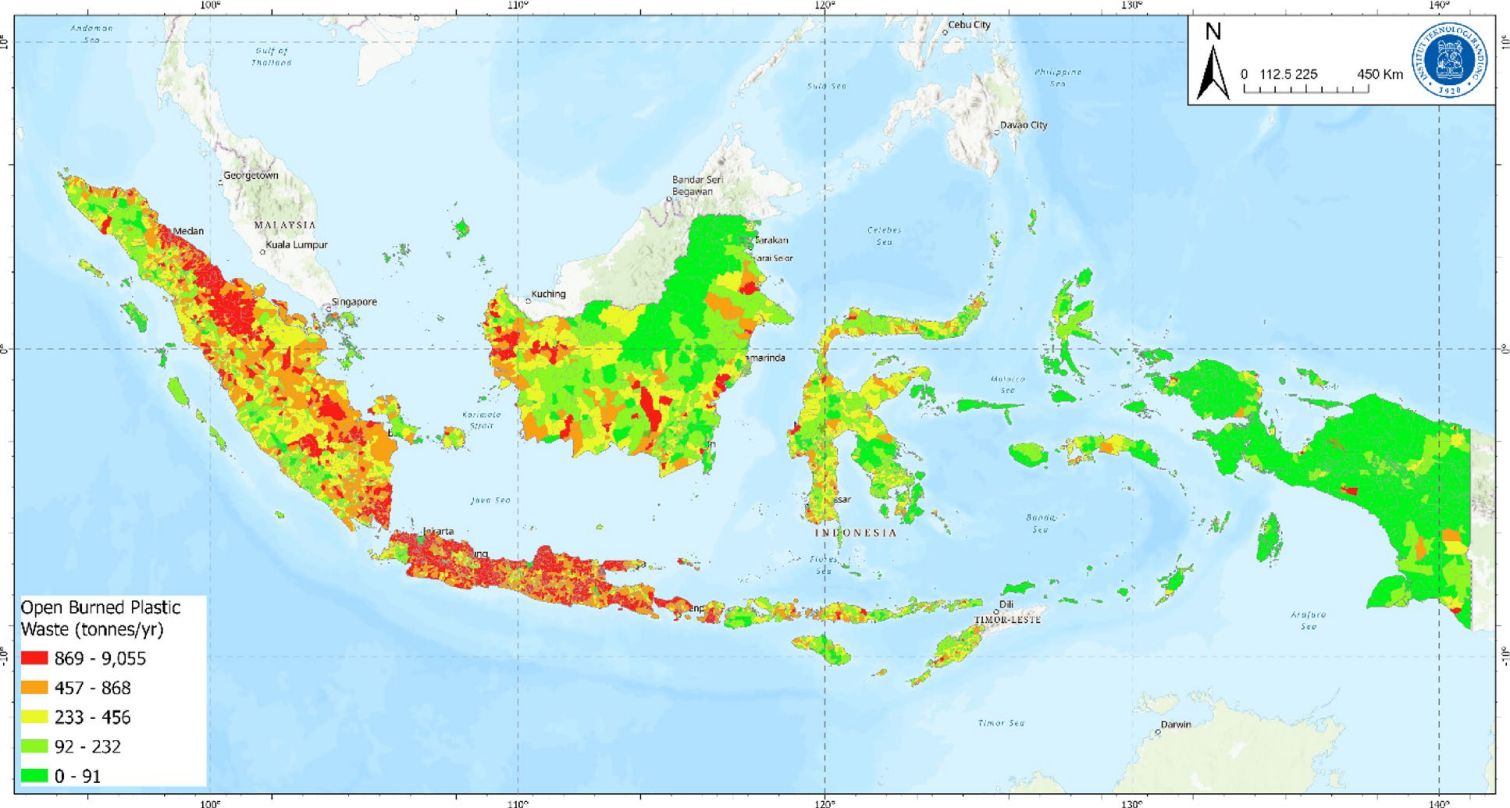
Openly burned plastic waste.

Open burning and burying of plastic waste at source categorized in this study as non-potential leakage because it does not immediately enter terrestrial or aquatic environments, open burning is the dominant practice. We estimate that approximately 4.15 ± 0.68 Mt of plastic waste are openly burned annually or 45% of total generation. This practice is predominantly found in rural areas or regions with limited access to formal waste management services. Meanwhile the buried plastic waste only 1% of total plastic waste generated or 0.12 ± 0.020 Mt per year. A map of open burning plastic waste is shown in Fig. 4.

## Discussion

### Monte Carlo model result, uncertainty and validation

This study verifies the model by comparing outputs through a sequential, dual-path analytical process. The verification in this study limited to the plastic waste generation, due to the limitation data of plastic fate from previous studies. First, the Monte Carlo model, derived from primary field sampling, is compared to the deterministic calculation based on National Waste Management Information System (SIPSN) baseline to identify systematic discrepancies. This stage highlights localized outliers where national data estimation averages fail to capture specific regional waste generation, revealing an inherent 8% overestimation trend in national reporting. Subsequently, a secondary comparison benchmarks the model against historical field studies to identify structural scope mismatches. Initial results indicate that when the model is compared to Urban areas with complete municipal total solid waste (TSW) sampling data, the Mean Absolute Percentage Error (MAPE) is approximately 10%. However, comparisons against rural sampling studies frequently focus on household solid waste (HHW), resulting in a high initial deviation of 73%. This divergence quantifies the unmeasured non-domestic waste fraction that limited-scope surveys systematically omit.

To resolve these disparities, the model integrates a distinct typology framework that categorizes each subdistrict (kecamatan) into Urban, Semi-Urban, and Rural classifications based on administrative data from the Directorate General of Population and Civil Registration^21^. The composition of each subdistrict determines its classification. Areas predominantly comprised of rural villages (desa) are categorized as rural, while those dominated by urban village (kelurahan) are designated as urban and mixed distributions are identified as Semi-Urban. Based on this typology, a stratified calibration was performed. A baseline calibration factor of 0.80 is applied to plastic waste generation in rural areas to mitigate systematic overestimation, while urban and semi-urban subdistricts maintain a factor of 1.0. Simultaneously, historical household only sampling data was normalized to a comprehensive total solid waste scope using non-domestic correction factors (0.45 for urban/semi-urban and 0.25 for rural areas) derived from previous sampling data of municipal level studies^22–25^. Detailed methodology and process is available in Supplementary Information 1.

The efficacy of this calibration is corroborated by regression analysis comparing uncalibrated and calibrated model outputs against the adjusted field benchmarks. Preliminary assessment of the uncalibrated model identified a systematic positive bias, most notably within low-density rural typologies such as the Gunungkidul (Yogyakarta) cluster (e.g., Purwosari, Panggang), where model exceeded adjusted empirical benchmarks by a factor of nearly two^26^. This confirms that the filling data coefficient is unsuitable with the limited consumption patterns of subsistence-based rural economies. However, the implementation of the 0.80 rural calibration coefficient significantly enhanced alignment. As evidenced by the regression analysis, calibrated data points converge significantly towards the line of unity, effectively mitigating the systemic positive bias. This predictive improvement is exemplified in the Pelaihari subdistrict in South Kalimantan, where the calibrated projection achieved a deviation of less than 10% relative to the benchmark. Furthermore, within rural typologies, the model retains a conservative positive margin (+ 20–30%), operationalized through a rural calibration factor of 0.8. This factor is set at a moderate level because most reference sampling studies were conducted in Gunungkidul, Yogyakarta, a relatively deep rural area characterized by arid karst terrain, structural poverty, and limited economic diversification^27^. If strictly calibrated to match Gunungkidul’s empirical profile, the factor would fall within 0.60–0.62.

**Fig. 5:**
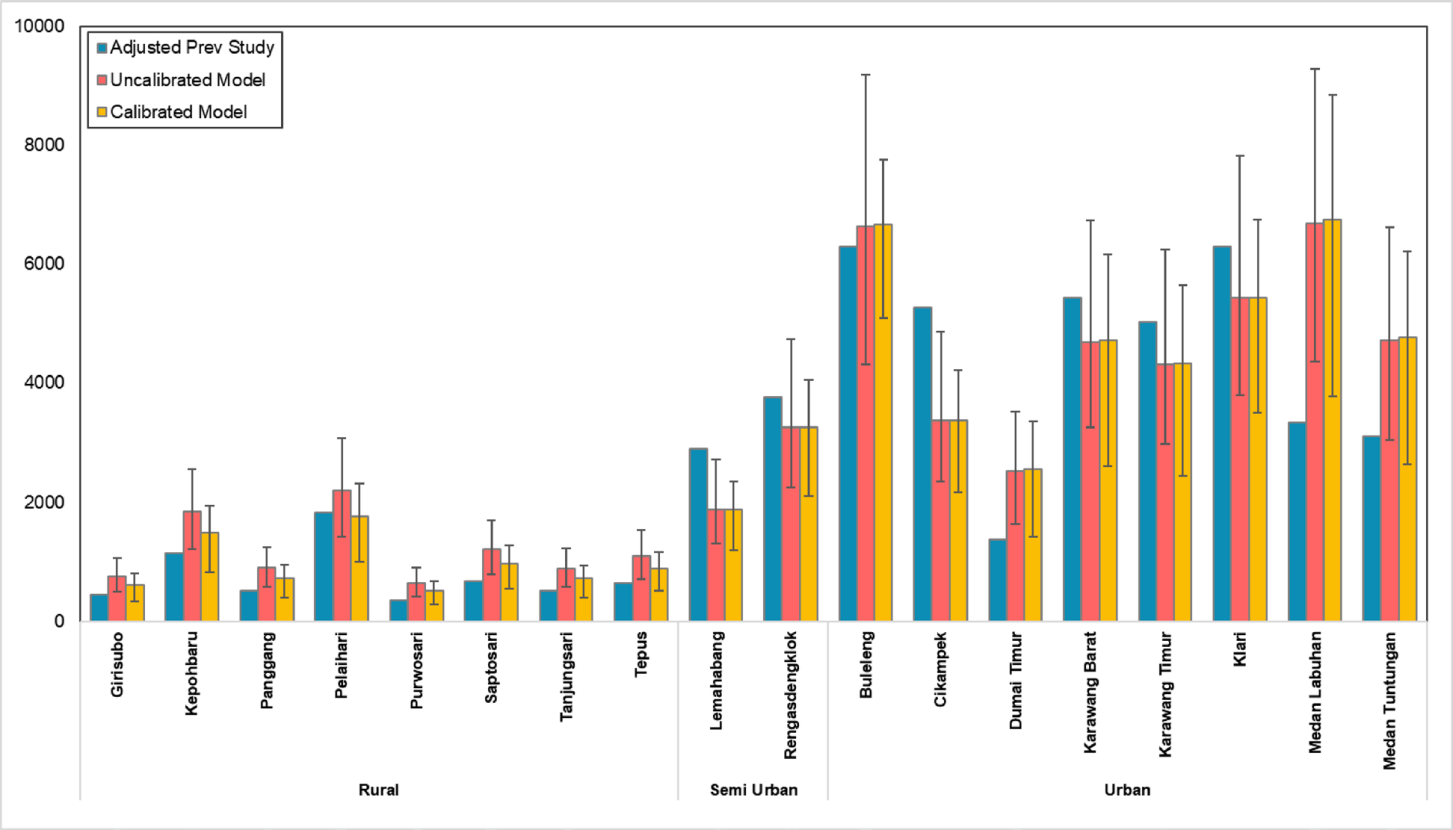
Comparison between previous studies, uncalibrated model and calibrated model.

However, the 0.8 factor is deliberately adopted to incorporate rural areas that are administratively classified as rural but are functionally influenced by nearby urban or industrial systems. This includes rural sub-districts located in regencies next to major metropolitan regions or urban areas where consumption patterns, market access, and waste generation characteristics tend to exceed those observed in deep rural contexts^28–30^. Thus, the selected factor ensures broader representativeness across heterogeneous rural profiles while avoiding over-adjustment toward deep rural baselines. The comparison between previous study, uncalibrated and calibrated model is shown in Fig. 5.

**Fig. 6:**
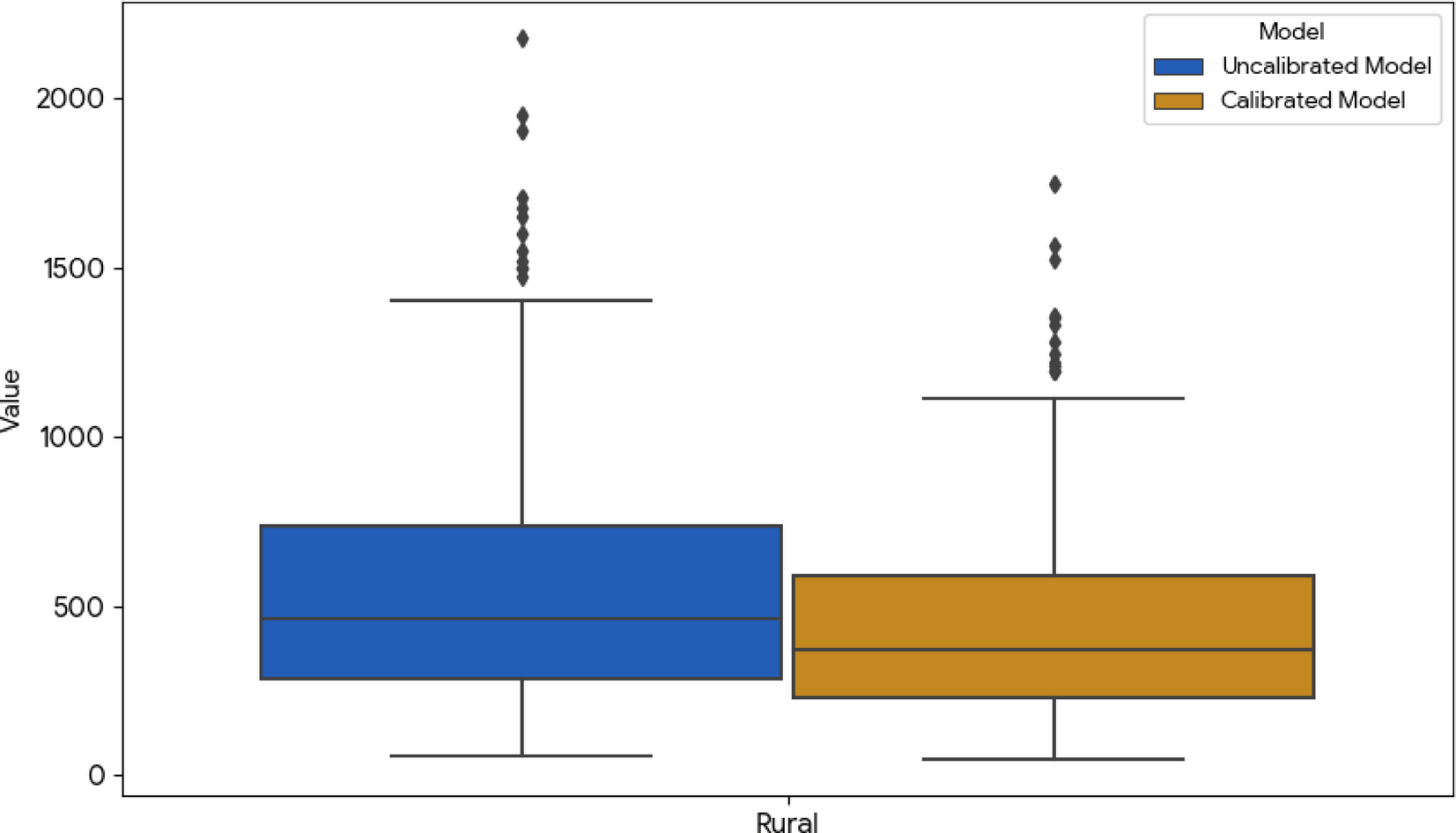
Comparison between uncalibrated model and calibrated model in rural areas.

The final tests confirm that the adjusted (calibrated) model is more reliable than the SIPSN data only. When compared to previous sampling studies, the calibrated model showed a low MAPE of 30% compared to uncalibrated model around 47%, proving it is accurate when properly set up. This process highlighted a clear gap in data quality, while urban data is generally good especially with complete sampling studies (domestic and non-domestic), the original rural estimates were consistently too high because they relied on simple population math that ignored the different conditions of rural life. By accounting factors like lower incomes and behaviour, the calibration reduced the total estimated plastic waste by 10%, dropping the national projection from 10.21 ± 1.61 Mt/year to 9.21 ± 1.52 Mt/year. Ultimately, this drop confirms that the original model overestimated rural plastic waste by failing to recognize that rural communities generate less waste. The result of comparisaon between calibrated model with uncalibrated model is shown in Fig. 6.

### Material flow analysis of plastic waste in Indonesia

Table 1 presents the estimated national solid waste generation and its management pathways, disaggregated into rural, urban, and semi-urban areas, with uncertainty ranges (±) reflecting statistical variability. Total national waste generation is estimated at 9.21 ± 1.52 Mt, of which rural areas contribute the largest share (5.84 ± 0.96 Mt), followed by urban areas (3.30 ± 0.55 Mt) and semi-urban areas (0.06 ± 0.01 Mt).

**Table 1:** Result of calibration and uncertainty analysis (tonnes/year).

Category	National (overall)	Rural	Urban	Semi Urban
Total generation	9,210,000 ± 1,521,000	5,842,000 ± 962,000	3,300,000 ± 547,000	64,000 ± 12,000
Managed	3,772,000 ± 622,000	1,315,000 ± 216,000	2,432,000 ± 405,000	24,000 ± 5,000
Buried	121,000 ± 20,000	105,000 ± 18,000	15,000 ± 3,000	1,000 ± 100
Open burning	4,154,000 ± 684,000	3,430,000 ± 565,000	692,000 ± 116,000	32,000 ± 6,000
Disposal to river	642,000 ± 106,000	545,000 ± 91,000	93,000 ± 17,000	4,000 ± 1,000
Disposal to land	521,000 ± 85,000	448,000 ± 73,000	69,000 ± 12,000	4,000 ± 1,000

**Fig. 7:**
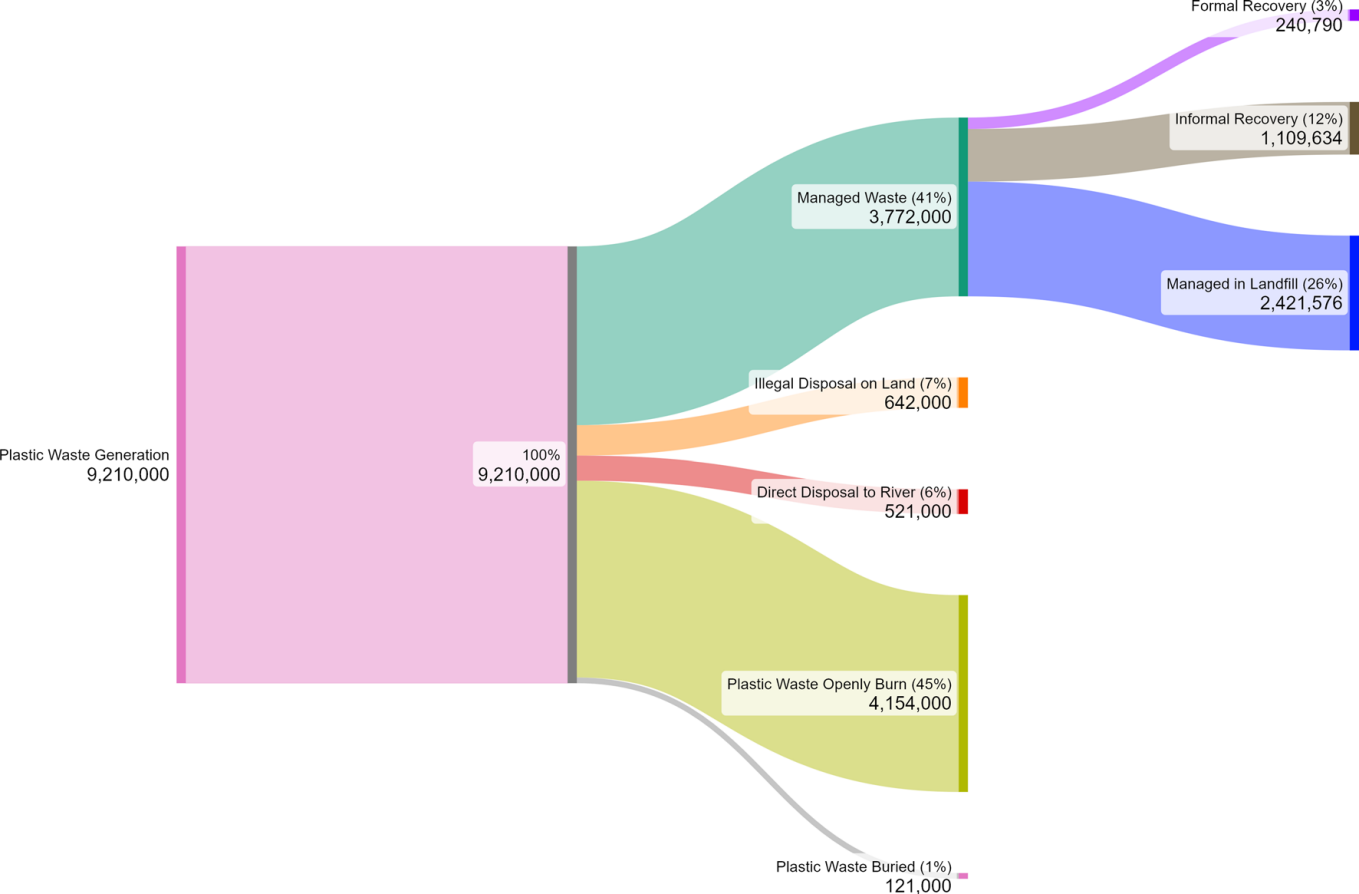
Material flow analysis of plastic waste in Indonesia (tonnes/year).

On the basis of the material flow analysis conducted, approximately 41% or approximately 3.77 Mt per year of plastic waste are managed in Indonesia. Urban areas account for the majority of managed waste (2.43 ± 0.41 million tons), indicating comparatively stronger service coverage and infrastructure concentration in cities. In contrast, rural areas manage only 1.32 ± 0.22 million tons, compared with formal recovery or facility-based management, the informal sector contributes more to waste recovery, with a percentage of 12%, and formal recovery at 3%. This condition is a characteristic phenomenon in many developing countries. This disparity is largely driven by the informal sector’s economic model (which operates as a profit-driven system) based on direct market incentives for recovering valuable materials, whereas formal systems are often underfunded municipal cost centers. The informal sector’s operational flexibility further enhances its efficiency, enabling it to conduct at-source separation before waste contamination occurs, operate with minimal overhead, and services low-income or inaccessible areas often bypassed by formal collection^31–33^. Moreover, approximately 26% or approximately 2.42 Mt of waste is managed or processed at landfill. Additionally, approximately 45% of plastic waste is openly burned and buried. This practice is overwhelmingly concentrated in rural areas (3.43 ± 0.57 million tons), highlighting systemic gaps in service provision and continued reliance on household-level disposal methods. The estimated amount of plastic disposed on land indicates that approximately 6% or 0.52 Mt per year of waste are disposed into terrestrial areas of on the basis of the analysis of waste generation data per subdistrict with data from the Riskesdas (2019)^16^. These findings highlight that open burning and illegal disposal remain the dominant fate of plastic waste across Indonesia, underscoring the urgent need for strengthening waste collection systems, improving treatment infrastructure, and fostering behavioral change to support the country’s marine plastic reduction goals. In total, this study reveals that annually, approximately 4.28 ± 0.70 million tonnes MPW that should not leak to the marine environment (e.g., openly burned and buried) and 1.16 Mt of MPW that could leak to the marine environment (direct disposal to land and river) are generated. Based on modeling conducted in Thailand, it is estimated that approximately 11% of plastic waste already present in terrestrial environments eventually enters water bodies via hydrological processes such as runoff and flooding^24^. These rates are highly sensitive to local geographical and climatic conditions. The overall material flow analysis of plastic waste in Indonesia is shown in Fig. 7.

Direct disposal into rivers represents 0.64 ± 0.11 million tons nationally, with rural areas still contributing the largest portion (0.55 ± 0.09 million tons). Although smaller in magnitude compared to open burning, this pathway is particularly critical from a marine debris and freshwater pollution perspective. Rural areas without adequate waste management infrastructure, is shaped by limited collection services and the normalization of practices such as burning, burying, or disposed the waste into waterways^20,34–37^. The practice persists because environmental responsibility is primarily oriented toward maintaining household and social cleanliness rather than protecting the wider natural environment, with rivers perceived as a practical means of removing waste from daily life^35^. Direct disposal into rivers results in plastic waste leakage into lakes and marine and marine environment^38^. While plastic waste entering rivers is a substantial finding, not all waste that enters rivers ultimately reaches the ocean. Previous studies states that only approximately 2% of the waste entering rivers ends up in the ocean^39^. Moreover, during extreme events such as floods, previous studies have shown a significant increase in plastic waste mobilization on land and in rivers but there is significant global disparity in the magnitude of mobilisation triggered by undefended flood events. While some country occurs a minimal rise of plastic transport, others face an escalation of up to 30 for instance in Vietnam^40,41^. Moreover, another study states that floods can increase plastic transport by up to 141 times, with approximately 30% of the annual plastic transport occurring within six days of flooding in Meuse River between Maastricht and Ravenstein^41^. However, plastic transport decreases significantly along rivers, with most plastic accumulating on riverbanks and floodplain areas, indicating that rivers serve as plastic reservoirs, with limited emissions to the sea^39,42^.

One of contribution of this research is the explicit quantification and constraint of uncertainty. The estimated 0.64 ± 0.11 Mt/year of direct disposal to rivers is comparable in magnitude to the estimate reported by Meijer et al. (2021) (0.82 Mt/year)^5^ and falls within the broader range presented by Lebreton & Andrady (2019) (0.37–1.73 Mt/year)^4^, but with a substantially narrower confidence interval. This improved precision results from the multilayer uncertainty framework applied, incorporating local data quality stratification (Q1–Q5) and region-specific management parameters rather than uniform national assumptions. However, the estimate is not directly comparable to global river-basin models. The 0.64 Mt/year represents only plastic waste directly disposed into rivers within the material flow analysis (MFA), excluding illegal land disposal (0.52 ± 0.09 Mt/year) and secondary mobilization via runoff. It should therefore be interpreted as a conservative, source-based baseline rather than a total riverine emission estimate. By isolating this leakage component, the study enhances transparency and provides an empirical lower-bound input for future hydrological or transport-based plastic leakage models.

### Plastic waste fate of each region in Indonesia

Table 2 shows that Java remains the dominant contributor to Indonesia’s plastic waste, generating approximately 5.37 million tonnes per year, or over half of the national total. This reflects its dense population and high economic activity. While Java records the highest proportion of collected waste (43%), it still relies heavily on open burning (45%), showing that unsustainable disposal practices persist even in the most developed regions. The region with the second-largest amount of plastic waste that is openly burned is Sumatera (0.79 Mt per year) where over 45% of waste is openly burned, posing serious risks to air quality and public health. Regions such as Bali–Nusa Tenggara and Sulawesi also exhibit substantial shares of open burning (45–49%) and leakage to rivers and land (15–19%), indicating that informal and environmentally harmful waste practices are still widespread across both urban and rural settings.

**Table 2:** Average of plastic waste fate between region in Indonesia.

Island/region	Collected waste	Buried waste	Openly burned waste	Disposed to river	Disposed to land	Total
	(tonnes/year)					
Sumatera	684,695	30,527	791,958	131,129	113,103	1,751,411
Java	2,327,724	67,481	2,438,035	328,061	208,695	5,369,997
Bali and Nusa Tenggara	171,303	6,292	246,477	42,138	35,404	501,614
Kalimantan	284,149	6,142	190,234	34,423	37,087	552,035
Sulawesi	232,184	8,085	301,355	57,364	70,180	669,168
Maluku and Papua	71,944	2,473	185,941	48,885	56,531	365,774
**Total**	**3**,**772**,**000**	**121**,**000**	**4**,**154**,**000**	**642**,**000**	**521**,**000**	**9**,**210**,**000**

Island/region	Percentage (%)					
Sumatera	39%	2%	45%	7%	6%	100%
Jawa	43%	1%	45%	6%	4%	100%
Bali and Nusa Tenggara	34%	1%	49%	8%	7%	100%
Kalimantan	51%	1%	34%	6%	7%	100%
Sulawesi	35%	1%	45%	9%	10%	100%
Maluku and Papua	20%	1%	51%	13%	15%	100%
**Total**	**41%**	**1%**	**45%**	**7%**	**6%**	**100%**

In contrast, Kalimantan has a relatively better collection rate (51%) but still experiences 34% open burning and noticeable direct river and land disposal. The situation in Maluku and Papua is particularly critical in terms of waste management infrastructure, with only 20% of the waste collected and a high proportion disposed directly to rivers (13%) and land (15%). However, in absolute terms, the total waste generated in these eastern regions (0.37 Mt per year) remains low compared with that in Java, meaning that despite the high percentage of illegal dumping, their overall environmental contribution is smaller.

While the plastic crisis is a global phenomenon, its burdens are disproportionately borne by middle-income countries, such as Indonesia, which find themselves at its epicenter. These nations are uniquely challenged, as accelerated economic growth and urbanization fuel escalating plastic consumption, which outpaces the development of their waste management infrastructure and financial capacity^43,44^. For example, in Indonesia, rural areas have become among the greatest contributors to mismanaged plastic waste. The primary reason for the high mismanagement of plastic waste in rural areas is the lack of waste management services. Rural areas often lack adequate infrastructure for collecting, transporting, and processing plastic waste, leading to widespread improper disposal in terrestrial environments^45^. This is exacerbated by the low percentage of waste management services in these areas. Waste management systems that do not cover various areas in a region lead to traditional open burning, which is often practiced in rural areas where waste collection services are unavailable^13^. Open burning is the most common method used in most rural areas in developing countries^12,46,47^, more common than recycling, open dumping and landfill. This is also the case in Indonesia, as reported in a study previous study, where approximately 50% of the waste generated in rural areas is openly burned by the local community^48^.

Our analysis yields a total plastic waste generation estimate of 9.21 ± 1.52 Mt/year, which is notably higher than the 8.14 Mt/year mean estimate reported in the recent global inventory by Cottom et al.^6^. This discrepancy stems fundamentally from the methodological divergence between bottom-up administrative aggregation and global predictive modeling. While previous study relies on machine learning regressions based on socioeconomic covariates (e.g., GDP, HDI) to infer local generation, our study integrates direct administrative data (SIPSN) and household surveys (Riskesdas). While global models often impute missing management data using regional averages or nearest neighbor algorithms, this research incorporates specific handling data unique to each regency, filling gaps with localized data (Riskesdas)^16^. This localized resolution is particularly evident in the quantification of openly burned plastic. This method is particularly evident in the quantification of openly burned plastic, which this study identifies at a mean of 4.15 Mt/year, around 2 times the 1.94 Mt/year mean estimated by Cottom et al.^6^. Results suggest that decentralized, more granular data analysis uncovers intensive mismanagement practices. Consequently, when compared to Thailand^24^, the 59% mismanagement rate identified here provides reflection of the localized infrastructure gaps and disposal behaviors unique to the Indonesian archipelago. The comparison between previous study and Thailand is shown in Table 3.

**Table 3:** Comparison of average mismanged plastic waste with previous study and Thailand.

Country`	Total plastic waste generated	Plastic emission (disposed to water environment, land and buried)	Plastic burned	Mismanaged plastic waste	Percentage of mismanaged plastic waste	Per capita (ton/year)
Indonesia (Mt/year) This research	9.21	1.28	4.15	5.43	59%	0.033
Indonesia (Mt/year)^6^	8.14	1.41	1.94	3.35	41%	0.030
Thailand (Mt/year)^49^	5.26	1.42	0.55	1.97	37%	0.079*

*based on calculation of population of Thailand in 2019^50^.

## Study limitation and future development

This study presents the first comprehensive, nationwide Material Flow Analysis (MFA) of plastic waste at the subdistrict (kecamatan) level in Indonesia. Despite this advancement, several important limitations must be acknowledged. Data availability and quality are uneven, with a substantial proportion of subdistricts relying on proxy or secondary data due to limited primary sampling, particularly in rural and eastern regions. Although uncertainty was addressed through a Data Quality Index, Monte Carlo simulation and calibration, validation was largely restricted to plastic waste generation, as empirical data on downstream fate (e.g., river transport, burial persistence, and open burning emissions) are scarce. Estimates of direct river disposal represent source-based baselines rather than fully coupled hydrological transport outcomes, and household-level disposal practices derived from survey data may introduce reporting bias.

Future research should prioritize expanded and systematic field sampling in rural and peri-urban areas to reduce reliance on calibration coefficients and provincial averages. Integrating this high-resolution MFA with hydrological and flood-based transport models would enable more accurate estimation of actual marine emissions rather than source-only leakage. Temporal analysis capturing seasonal variability, particularly during monsoon periods, would further improve understanding of episodic plastic mobilization. Strengthening administrative reporting systems, refining informal sector quantification, and incorporating behavioral and socioeconomic analyses will also be critical to enhance model precision and support more targeted, evidence-based waste management and environmental policy interventions. The leakage source result produced here could be coupled with hydrological transport models to trace the fate and movement of plastics through river networks to the ocean, building on existing studies of plastic transport pathways^17,18^. The GIS framework can also be enhanced with predictive modeling by integrating additional geospatial variables such as poverty indices and road networks to better anticipate areas of high leakage risk. Finally, the hotspot maps generated in this study can guide targeted, field-based waste characterization efforts in priority to refine key parameters and improve the accuracy of future national models.

## Methods

### Data collection and assumptions for the MFA

Data collection for this research involves the utilization of secondary data sourced from the Indonesia National Waste Management Information System (SIPSN) website and relevant government departments across Indonesia. The data utilized include information necessary for calculating the material flow of waste from its sources to final processing, encompassing equivalent waste generation, waste collection, reduction, transportation, and final processing at landfill sites. Additionally, population data are incorporated to ascertain population figures and density across various regions. These data include population numbers at the subdistrict level and population density data obtained from national statistical agencies such as the Central Statistics Agency (BPS) and the National Directorate of Population and Civil Registration from 2023^21^.

Data on waste generation comprise both per capita and total waste generation figures sourced from the SIPSN, IKPLHD (Regional Environmental Management Performance Information) strategic plans, and relevant government departments, with composition percentages, particularly plastics, and the SIPSN. Information regarding waste management at its sources encompasses data on waste managed by the government, open burning, burying, illegal dumping, and disposal into rivers, sourced from the National Basic Health Research (Riskesdas) published in 2019^16^. Furthermore, data on waste management facilities and informal sectors, temporary waste collection facilities (TPS/transfer stations) related to waste recycling and the informal sector include figures on waste reduction through TPS3R (Waste Bank), middlemen and aggregators sourced from the Ministry of Public Works and Public Housing (PUPR), SIPSN from 2020 to 2023, and relevant government departments. We are also introducing the rural, semi urban and urban areas using analysis of data from Directorate General of Population and Civil Registration of Indonesia to address the spatial heterogeneity of waste generation across Indonesia,

###  Data preprocessing and imputation

The data preprocessing and imputation procedures (consists of data selection, cleaning and filling) was conducted in this study primarily to address missing entries and datasets that prone to bias compared with actual data. The process is outlined as follows:

- Removing duplicate data.
- Data are selected on the basis of data age, with the latest data considered the most reliable.
- To assume the necessity of per capita waste generation data, empty data for each region’s waste generation will be filled with average per capita waste generation data within the same province and the same type of administrative area (city or district). In 2022 and 2023, only approximately 309 of 514 municipalities submitted waste generation and management data to the SIPSN^15^.
- The data from Riskesdas (2019) are assumed to be the baseline of managed waste in this study, if SIPSN waste management performance exceed a 30% difference compared to the Riskesdas 2019 baseline, the Riskesdas is retained as the correct value. The 30% improvement threshold is applied as a plausibility filter to reduce the risk of overestimation in administrative waste management data. This threshold is based on the difference between international cases of rapid system reform in other countries and the observed trends in Indonesia. Evidence from countries that experienced major improvements in waste management shows that increases of 25–50% percentage points (pp) represent the upper limit of progress and typically occur only under exceptional conditions, such as large-scale government investment or major institutional reform for example in China (+ 25 pp, 2008–2014)^50^, Bosnia and Herzegovina (+ 41 pp, 2008–2017) and Morocco (+ 52 pp, 2008–2021)^51^. In contrast, data from Indonesian regions show gradual and incremental changes. High-performing urban areas such as Yogyakarta City (≈ + 2.63 pp/year)^52^, and Pematangsiantar (≈ + 2.83 pp/year)^53^, demonstrate steady but moderate annual improvements, while more rural municipalities such as Sleman and Bantul show minimal change (< + 0.1 pp/year)^52^. Within this context, a difference greater than 30% between self-reported SIPSN data and the independent, household-based Riskesdas (2019) baseline would imply an annual increase of more than 6.0 pp/year. Such a rate of change is unlikely to reflect actual infrastructure expansion and is more likely associated with inflated administrative reporting coverage. Therefore, the 30% threshold is used as a reasonable control measure, with Riskesdas 2019 serving as a reference baseline to ensure that the analysis reflects actual household waste disposal behavior rather than changes in reporting practices.
- Due to the scarcity of local waste characterization studies, this study utilized the national average plastic composition of 19.03% to impute missing values for municipal, commercial, and industrial waste streams. The use of national aggregate data as a proxy for local waste generation and composition is a standard and necessary approach in large-scale plastic pollution modeling when local data is unavailable. This imputation method aligns with established global baselines set by the World Bank^2,14^, and has been widely adopted in foundational plastic transport models, to ensure consistent estimation across heterogeneous geographic areas^3,5,7^.

### Material flow analysis

**Fig. 8:**
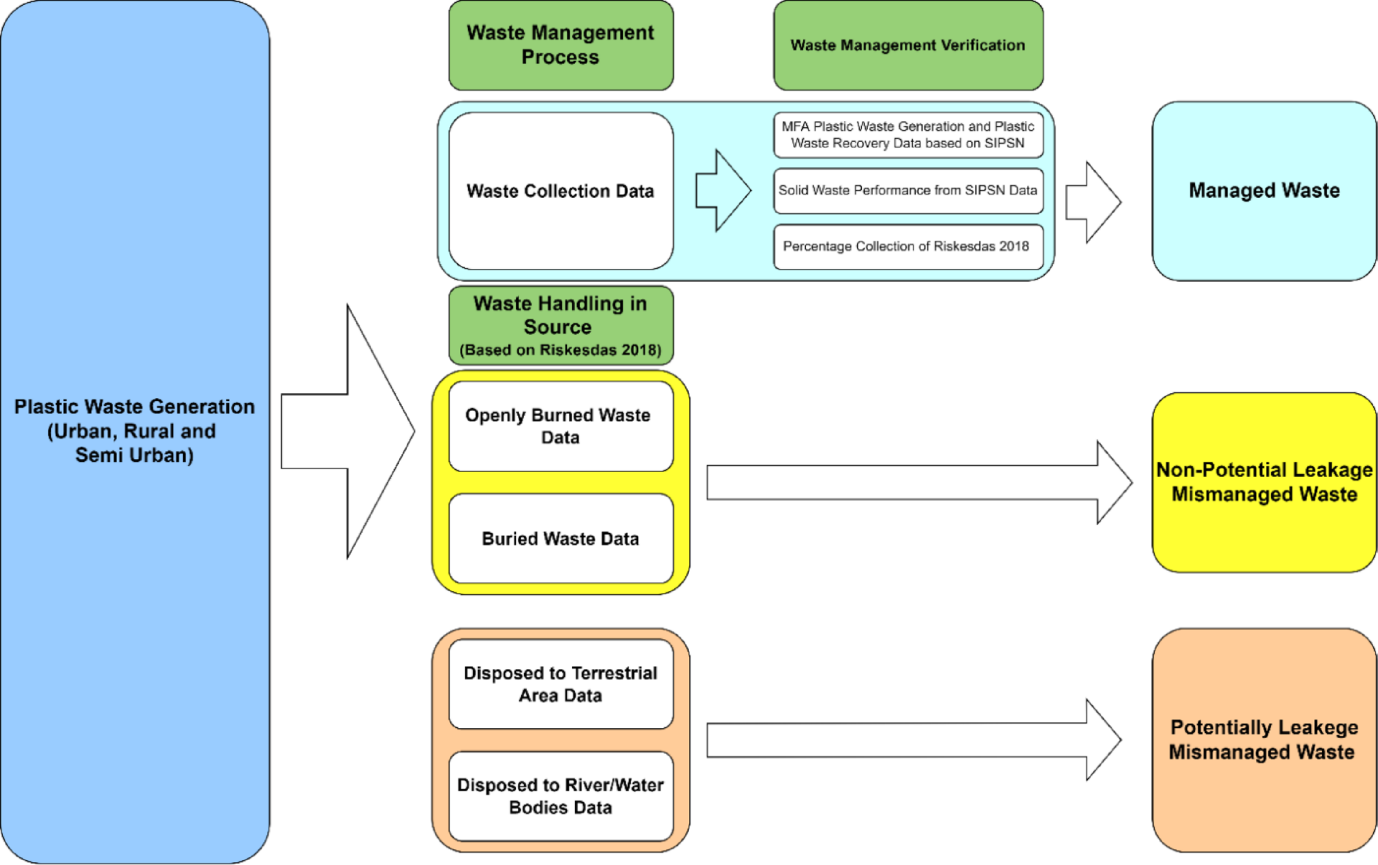
Framework of MFA for plastic waste in Indonesia.

The initial stage of this research involves estimating the potential mismanaged plastic waste (MPW) from sources by considering waste management and disposal facilities. This calculation aims to understand the existing waste management system, waste generation, and available data in Indonesia, as well as to estimate the potential waste that may become MPW both non-potentially and potentially leakage MPW, which could lead to leakage into the environment. This method builds upon and develops approaches used in other studies^2–6,54^. This analysis is conducted via the principles of material flow analysis (MFA) combined with spatial data processed via GIS software to determine the potential unmanaged plastic waste in each subdistrict. MFA is a quantitative analytical method that uses the mass balance principle to measure the flows and stocks of materials within a system defined in space and time. All inputs into a system are equivalent to all outputs, including the accumulation or depletion of material stocks inside the system. The framework of the MFA in this paper is shown in Fig. 8.

### Geospatial analysis

After the completion of the MPW analysis for each subdistrict in Indonesia, ArcGIS software was used to create a map displaying potential MPW indices at the subdistrict (kecamatan) level. This process involves incorporating various inputs, such as subdistrict boundary maps, plastic generation and MPW per capita data derived from material flow analysis (MFA). Additionally, exploration of previous research data on waste generation and composition at the subdistrict (kecamatan) level will be conducted to enhance the analysis. Combining population and waste generation data per capita will enable the determination of the MPW potential for each subdistrict (kecamatan), facilitating the identification of areas with the highest potential for MPW accumulation. Finally, quantile classification within ArcGIS software allows the creation of a five-class index, categorizing MPW potential into levels ranging from very high to very low, thus providing a comprehensive understanding of the MPW distribution across Indonesia at the subdistrict level.

### Uncertainty analysis

The uncertainty in the plastic waste material flow analysis (MFA) was quantified via Monte Carlo simulation (MCS) with 10,000 iterations using Python with Panda and NumPy library. The methodology constructs a multilayer uncertainty framework for plastic waste material flow analysis (MFA) by first deconvolving uncertainty into its fundamental components^55^. This approach models all uncertain inputs via lognormal distributions, which ensures that all sampled values (such as waste generation) remain nonnegative. A core feature of this methodology is the separation of uncertainty into two types: systematic (global) uncertainty, which applies a single random bias to all 514 municipalities simultaneously in a given run (e.g., a systemic data collection bias), and local uncertainty (uncertainty, which applies an independent random factor to each municipality to account for local errors and natural variability).

The input parameters are characterized by combining baseline data with these uncertainty factors. For waste generation, the baseline per capita value for each municipality is multiplied by both a systematic factor coefficient of variation (CV = 0.10) and a local factor (CV ranging from 0.10 to 0.50). Waste management fractions (e.g., collected, open burning, river disposal) are treated as compositional data. In each iteration, their baseline values are multiplied by a systematic factor (CV = 0.10) and a local factor (CV = 0.20), and then the resulting fractions are normalized to ensure that they sum to 100%, thus maintaining mass balance. Other parameters, such as population and the national average plastic percentage, are also assigned small, independent CVs to represent their respective uncertainties. The model is run for thousands of iterations, sampling from each parameter’s defined distribution in every run^6^. This process generates a full probability distribution for all key outputs, such as the total Mismanaged Plastic Waste (MPW) for each region and the national total. The final results were reported not as single point estimates but as a median (50th percentile) value accompanied by a 95% confidence interval (CI), which captures the range of plausible outcomes^56^. The uncertainty numbers are shown in Table 4.

**Table 4:** Parameters in the Monte Carlo analysis.

Parameter	Parameter	Value
Simulation runs	Monte Carlo	*n* = 10,000
Uncertainty of systematic generation	Global (CV)	0.10 (10%)
Uncertainty of systematic management	Global (CV)	0.10 (10%)
Uncertainty of local management	Local (CV)	0.20 (20%)
Uncertainty of local generation (Based on the data qualities)	Local (CV)	10% − 50%

### Calibration and validation data

The verification framework for this material flow model follows a sequential identification process that begins with a dual-path comparative analysis. First, Monter Carlo model derived from primary field sampling is juxtaposed against the deterministic National Waste Management Information System (SIPSN) baseline to identify systematic discrepancies. This stage highlights localized outliers where national averages fail to capture specific regional consumption intensities. This is followed by a comparison between the uncalibrated model and secondary data from previous studies to identify structural scope mismatches.

To resolve these disparities, a stratified calibration is performed based on subdistrict typologies (Rural, Urban and Semi Urban) identified through data from the Directorate General of Population and Civil Registration^21^. The administrative composition of each subdistrict determines its classification, areas predominantly comprised of rural villages (desa) are categorized as rural, while those dominated by urban urban villages (kelurahan) are designated as urban. In cases where the administrative distribution is equal, the subdistrict is identified as semi-urban. Based on this typology, a baseline calibration factor of was 0.8 is applied to the plastic waste generation in rural areas to mitigate systematic overestimation, while urban and semi-urban subdistricts maintain a factor of 1.0. The complete information of this process is available at Suplementary Information 1.

## Supplementary Information

Below is the link to the electronic supplementary material.

Supplementary Material 1

Supplementary Material 2

## Data Availability

The details method and result of the model is available in Suplementary Information. The raw data that support the findings of this study are available from the corresponding author, E.S, upon reasonable request.
