# Supplementary material for: Mapping mismanaged plastic waste in Indonesia: subdistrict-level analysis through material flow from sources to the environment
Source: Sci Rep. 2026 Mar 13;16:13341. doi: 10.1038/s41598-026-41849-w (PMC13106676; doi:10.1038/s41598-026-41849-w)
Supplement: Supplementary file 1 — Supplementary Material 1 [file 41598_2026_41849_MOESM1_ESM.docx]

Supplementary Material

**Mapping Mismanaged Plastic Waste in Indonesia: Subdistrict-level Analysis through Material Flow from Sources to the Environment**

# Attar Hikmahtiar Ramadan^1^, Emenda Sembiring^2*^, Benno Rahardyan^2^, and Hadi Kardhana^3^

1Doctoral Program of Environmental Engineering Study Programme, Faculty of Civil and Environmental Engineering, Institut Teknologi Bandung, Bandung 40132, Indonesia

2Air and Waste Management Research Group, Faculty of Civil and Environmental Engineering, Institut Teknologi Bandung, Bandung 40132, Indonesia

3Water Resources Engineering Research Group, Faculty of Civil and Environmental Engineering, Institut Teknologi Bandung, Bandung 40132, Indonesia

*Corresponding Author: Emenda@itb.ac.id

1. **Methodology**

A critical methodological step in this research is the refinement of "mismanaged plastic waste" (MPW) to isolate the specific fraction relevant to environmental leakage. We categorize MPW on the basis of its mobilization potential—that is, whether it can be transported from its disposal site by environmental forces such as hydrological runoff.

1. Potential-Leakage MPW: This category includes all MPW fractions that are available for mobilization and form the input for our transport model. It is operationally defined as (a) plastic littered onto the terrestrial environment and (b) plastic directly disposed of into water bodies.
2. Nonleakage MPW: This category includes mismanaged pathways that, while environmentally harmful, remove plastic from the surface transport system. For the purpose of this model, it is defined as (a) unmanaged buried waste and (b) openly burned plastic waste.

This distinction is fundamental. This allows us to accurately quantify the true pool of plastic waste available to be mobilized by runoff, preventing the overestimation that would result from including nonavailable (buried or burned) waste in a hydrological transport model.

The analysis is conducted by combining the material flow analysis (MFA) principle with spatial data processing in a GIS environment. This method builds upon and develops approaches used in previous studies [1–6]. This methodological combination is essential for two key reasons:

1. To quantify "how much" (MFA): The MFA is the accounting framework used to track the mass balance of plastic from its point of generation through the entire waste management system. This allows us to quantify the total volume of waste that exits the formal collection and disposal system, creating the total "Mismanaged Plastic Waste" pool.
2. To pinpoint "Where" (GIS): A simple MFA provides a nonspatial number (e.g., "X tons of MPW for Regency Y"). This is insufficient because leakage risk is fundamentally spatial. By integrating the MFA data with GIS data, we can disaggregate this total MPW down to the subdistrict (kecamatan) level and assign it to specific locations on the basis of population density, land use, and proximity to transport pathways such as rivers.

In this study, data on waste generation for each district/city are incomplete and not always available annually, according to the SIPSN and related agency data. Therefore, the current study assumes that per capita waste generation per day remains constant from 2020–2023, but in this research, we also measure the data uncertainty specific to the data age and data sources. This considers that the annual waste generation in a city/district will be divided by the population data for the same year (e.g., generation in city A in 2022 will be divided by the population of city A in 2022); thus, the generation value changes relative to the population.

For unavailable data on waste management facilities, waste handling data from the SIPSN for each district are included. If that is also missing, data from the Riskesdas 2018 analysis (percentage of waste collected) will be used. On the basis of the data hierarchy and timeliness, the data selection priority is:

1. Data on waste management (from source, transport, recovery, and final processing).
2. Data on waste management performance (handling and reduction) from the SIPSN.
3. Data from analysis via Riskesdas 2018 (the most complete data for handling at the source).

In ArcGIS, calculation and layering processes are performed to determine the MPW potential for each subdistrict in Indonesia. After the MPW data are analyzed for each region, this study displays an MPW potential index map at the subdistrict level. Currently, the available data in Indonesia are at the district/city level. To create an MPW map at the subdistrict level, this stage uses the following approach: a. Input various data for grid creation in ArcGIS, including (i) administrative boundary maps (districts and subdistricts) and (ii) population density maps. b. MPW per capita data (derived from district-level MFA data) are divided by the district's population. c. In addition to SIPSN data, data from previous research regarding waste generation and composition at the subdistrict level will be explored. d. Combining waste facility data and waste management characteristics in urban and rural areas on the basis of Riskesdas data (handling at source and facility conditions). e. Population density and per capita waste generation data are combined to derive the potential MPW for each subdistrict, allowing for the determination of high-potential areas via GIS.

The index consists of 5 classes: very high, high, moderate, low, and very low. The index range is determined after the MPW calculations are complete. This index is created via the quantile method available in ArcGIS. The quantile is used to divide the dataset into equal parts on the basis of specific variable values. In the GIS context, quantile classification is used to divide classes so that the total number of features (e.g., shapefiles, waste generation data) is approximately the same. This classification type is useful for showing rankings and ordinal data [7,8].

**The Rationale for Data Fusion**

A primary challenge in Indonesian waste modeling is that no single government agency provides a complete dataset. The data are fragmented, collected for different purposes, and have significant, well-known gaps (such as the informal sector).

Therefore, this research employs a data fusion approach, combining and cross-validating data from multiple sources to build a comprehensive model that overcomes the limitations of any single source.

1. Dukcapil Nasional (Directorate General of Population and Civil Registration): Waste generation is a direct function of the population. To move from the regency (kabupaten) level to the subdistrict (kecamatan) level, we need the most accurate, high-resolution, and current population data available. Dukcapil's semester-based registry data constitute the definitive population source, making it far more precise and timely than projections based on the 10-year census. These data are the core variables used to spatially disaggregate the total waste generated. Based on this we retrieve rural, semi urban and urban data.

Role: Provides the primary demographic driver for the entire model.

1. Primary Sampling Data: This data is the "ground truth" data, collected directly from the field through rigorous, site-specific Waste Analysis and Characterization Studies (WACS). Unlike the other sources, which rely on reporting, estimation, or perception, primary sampling provides the most accurate, empirical measurement of actual waste generation rates, composition, and density.

Role: Serves as the primary benchmark for comparison and model validation. While too expensive and time-consuming to conduct nationwide, these studies (where available for specific cities or regions) are essential for:

1. Calibrating the model's baseline assumptions (e.g., validating the per-capita generation rates reported in SIPSN).
2. Assessing the accuracy of the plastic waste composition percentages used in the model.
3. Providing a quantitative basis for the uncertainty analysis by showing the real-world variance between "reported" data and "sampled" data.
4. SIPSN (Ministry of Environment - KLHK) & Local Environmental Agencies (DLH): These sources provide the top-down baseline for the formal waste system. The SIPSN contains official, self-reported data from regencies on total waste generation, formal collection rates, waste composition, and the amount of waste processed at formal facilities (TPA, TPS3R, etc.). Local DLH data can supplement or validate these figures at a more granular level.

Role: Defines the inputs (generation) and formal management pathways of the MFA.

1. RISKESDAS (Ministry of Health): These national socioeconomic and health surveys are the keys to understanding unmanaged pathways. SIPSN tells us what is collected, but it does not tell us what happens to the waste that is not. RISKESDAS and SUSENAS survey households directly about their behavior, providing critical data on the methods of unmanaged disposal (e.g., "What does your household do with its waste? ").

Role: Provides the behavioral data needed to split the total unmanaged waste into specific pathways: littered, dumped to water, burned, or buried. This directly fills the informal sector gap left by the SIPSN.

1. BPS (Statistics Indonesia) & BIG (Geospatial Information Agency): These agencies provide the spatial and demographic backbone of the model.

Role: The BPS provides population data at the subdistrict or village level, which is the primary proxy used to disaggregate waste generation. BIG provides essential GIS layers, such as administrative boundaries (kecamatan), river networks, coastlines, and land use maps.

A crucial component of this research methodology is the formal quantification of uncertainty. Given that the model relies on a fusion of data from multiple agencies—each with different collection purposes, methodologies, and limitations—it is essential to move beyond a single deterministic result. We therefore develop a data quality index (DQI) to formally assess and normalize the uncertainty associated with the primary data inputs. This DQI will be used to create final "confidence data" that accompany the Mismanaged Plastic Waste (MPW) results. This approach allows policymakers and stakeholders to see not only where the leakage hotspots are but also how reliable the data are for that specific area.

This data quality index is a normalized score. The score is a composite value based on several key criteria that define the "condition" of the data. These criteria include its timeliness (e.g., data from the last two years versus data older than five), its methodology (e.g., direct measurement from a weighbridge versus estimations based on population), its completeness (e.g., all required fields present versus significant gaps), and its spatial resolution (e.g., data native to the kecamatan level versus kabupaten-level data that must be disaggregated).

This DQI framework is applied to each data source differently. Population data from Dukcapil are generally of high quality, as they are timely, registry-based, and spatially explicit and would therefore receive a high DQI. In contrast, the data from the SIPSN and local DLH offices are the most variable; a regency that provides complete, recent data based on measured weighbridge inputs would receive a high DQI, whereas a regency that provides incomplete estimates would receive a very low DQI. Similarly, behavioral data from RISKESDAS or BPS are methodologically strong due to statistical sampling, but their score may be moderated if the data are only representative at the kabupaten level, thus introducing uncertainty when applied to a kecamatan.

1. **Scope**

This research encompasses a national-scale quantitative assessment of Mismanaged Plastic Waste (MPW) in Indonesia, with a final analytical resolution at the subdistrict (kecamatan) level. The focus of this study is thematically constrained to Potential-Leakage MPW, which is operationally defined as plastic that is disposed in terrestrially area directly disposed of in water bodies. This method specifically excludes mismanaged pathways considered "nonleakage" (i.e., open burning and unmanaged burial) to accurately quantify the specific pool of waste available for hydrological transport. Methodologically, the scope involves a material flow analysis (MFA) combined with GIS spatial processing, built upon a data fusion approach. This approach integrates national demographic data (Dukcapil), formal waste system data (SIPSN, DLH), behavioral data (RISKESDAS), and geospatial framework data (BIG, BPS) for the 2020-2023 period. A core component of the scope is the formal quantification of uncertainty via a data quality index (DQI). The final deliverable is a high-resolution potential MPW index map for all subdistricts, which serves as the primary input for subsequent environmental transport modeling.

1. **Review of Data Resources**

Data collection in this study uses secondary data obtained from the National Waste Management Information System (SIPSN) website and related government agencies throughout Indonesia. The data used in this stage include data for calculating the material flow of waste from the source to final disposal. This encompasses data on waste generation (domestic and nondomestic), waste collection, waste reduction, waste transportation, and final processing at landfills (TPA) [9]. Additionally, population data (from Dukcapil Nasional) are used to determine the population and population density in each region. The various data required for this study are detailed in Table S.1.

**Table S.1.** List of Data Collected

| **Category** | **Parameter** | **Description and Data Source** |
| --- | --- | --- |
| Data and Regional Maps | Map of Indonesia | Geospatial Information Agency (BIG)  URL: <https://geoportal.big.go.id/#/viewer> |
| Indonesian Population Data | Population at the subdistrict level | Dukcapil Nasional (National Directorate General of Population and Civil Registration)  URL: <http://gis.dukcapil.kemendagri.go.id/arcgis/rest/services> |
|  | Rural and urban population |  |
|  | Population density map |  |
| Waste Generation Data | Waste generation (per capita and total) | 1. SIPSN, IKPLHD (Regional Environmental Management Performance Information.), City/Regency Strategic Plans, and Related Agencies   URL:   1. <https://portal-sipsn.kemenlh.go.id/data/timbulan-sampah> 2. <https://portal-sipsn.kemenlh.go.id/data/komposisi-sampah> 3. Field Sampling |
|  |  |  |
|  | Waste composition (percentage of plastic) |  |
| Data on Waste Handling at Source | Data on waste burned | Riskesdas (Basic Health Research) 2018  URL: <https://layanandata.kemkes.go.id/katalog-data/riskesdas/ketersediaan-data/riskesdas-2018> |
|  | Data on waste buried |  |
|  | Data on waste disposed indiscriminately |  |
|  | Data on waste disposed into rivers |  |
| Data on waste collection services | Collected Waste Data | 1. SIPSN   URL:<https://portal-sipsn.kemenlh.go.id/data/capaian>   1. Riskesdas (Basic Health Research) 2018   <https://layanandata.kemkes.go.id/katalog-data/riskesdas/ketersediaan-data/riskesdas-2018>   1. IKPLHD 2. Field Sampling |
|  | Government/Agency Reports |  |
|  | Amount of waste collected by informal sector |  |
|  | Data on self-managed waste by community |  |
| Data on temporary collection facilities (TPS/Transfer Station) | Data of TPS/TPS3R (Reduce, Reuse, Recycle Waste Management Site) | SIPSN  URL:   1. <https://portal-sipsn.kemenlh.go.id/fasilitas/tps3r-pdu-itf> 2. <https://portal-sipsn.kemenlh.go.id/fasilitas/sumber-energi> 3. <https://portal-sipsn.kemenlh.go.id/fasilitas/tpa-tpst> 4. <https://portal-sipsn.kemenlh.go.id/fasilitas/sektor-informal>   Data from Ministry/Government/Related Agencies  Previous research data |
|  | Capacity of TPS/TPS3R |  |
|  | Spatial distribution of TPS/TPS3R |  |
| Data on recycling and informal sector | Amount of waste reduced by TPS3R | 1. Ministry of Public Works and Housing (PUPR) 2. SIPSN and related agencies   URL:   1. <https://portal-sipsn.kemenlh.go.id/fasilitas/sektor-informal> 2. <https://portal-sipsn.kemenlh.go.id/fasilitas/bank-sampah> |
|  | Amount of waste reduced by Waste Banks |  |
|  | Amount of waste reduced by informal sector (scavengers, stalls, collectors) |  |
| Potential Leakage Data | Leakage data from each waste facility | Previous research and Literaturu |
| Landfill (TPA) Data | Sanitary Landfill | 1. Data from Ministry of PUPR 2. Data from SIPSN   URL:  <https://portal-sipsn.kemenlh.go.id/fasilitas/tpa-tpst> |
|  | Controlled Landfill |  |
|  | Open Dumping |  |

Waste management data is one of the most critical elements of this study, serving as the primary input for identifying waste sources with the potential for environmental leakage in each region. This data encompasses waste treatment, management, reduction, and final disposal processes. The data required for this research includes:

1. Data on waste generation, composition, and management at waste facilities.
2. Data on waste treatment and management at the source (both domestic and non-domestic).
3. **SIPSN**

SIPSN, which stands for Sistem Informasi Pengelolaan Sampah Nasional, is Indonesia's official National Waste Management Information System. It is a web-based platform managed by the Ministry of Environment and Forestry (KLHK) and established by Ministerial Regulation No. 6 of 2022. The primary purpose of the SIPSN is to create a single, centralized database by collecting, integrating, and publishing waste management data from all 514 regencies and cities across the country. This system is designed to track key metrics such as total waste generation, waste composition (e.g., plastic, food, paper), the amount of waste successfully reduced through 3R activities, and the amount of waste handled at formal facilities. Ultimately, the SIPSN serves as the government's primary tool for monitoring national progress toward policy goals, such as those in the National Waste Management Policy (Jakstranas), and for providing public access to waste data. The data available on this website includes:

- 1. Waste Sources: Data on the origin of waste (e.g., households, industry, and other non-domestic sources).

1. Waste Generation: Data on the total amount of waste generated (per day and per year).
2. Waste Composition: Data on the types of waste and their respective percentages (e.g., organic, plastic, paper, metal, other).
3. Waste Management Facilities: Data on facilities used to manage waste, such as TPST (Integrated Waste Treatment Facility), TPA (Landfill), TPS3R (3R Waste Management Facility), PDU (Recycling Center), Waste Banks, ITF (Intermediate Transfer Facility), and others.
4. Other Information: Other data related to waste management.

However, not all regencies/cities deliver this database. Since its inception in 2018, there has not been a single year with complete data for all regencies/cities. In 2022 and 2023, only approximately 309 regencies/cities submitted data on waste generation and management.

The main strengths of the SIPSN lie in its centralization and transparency. Before its implementation, waste data were fragmented, inconsistent, and difficult to compare, as each local government office (Dinas Lingkungan Hidup) used different reporting methods. SIPSN provides a standardized format, allowing for national-level aggregation and analysis. Furthermore, because the portal is publicly accessible, it fosters transparency and accountability, enabling researchers, NGOs, and the public to access official government statistics. These data are essential for the central government to monitor national performance and identify regions that are falling behind targets.

However, the SIPSN suffers from several significant weaknesses, with the most critical being the quality and reliability of its data. The system operates on a "waste in, waste out" principle, meaning that its accuracy is entirely dependent on the quality of the data self-reported by each local government. Many regional offices lack the budget, technical capacity, or equipment (such as weighbridges at landfills) to conduct accurate waste surveys, often relying on estimates. This leads to inconsistent and incomplete reporting, with data from many of the 514 regencies often missing, which creates significant gaps in the national statistics. The previous highlights that SIPSN reporting is often poorly coordinated, leading to irregularities and inconsistencies that make the data unreliable for policy-making. Historically, reporting has been unstable, with participation crashing from 276 regions in 2020 to just 146 in 2021, indicating that reliability depends more on regional administrative priority than actual waste flow. While Ministerial Regulation No. 6 of 2022 improved participation, a substantial gap remains, with 109 local governments still failing to report and 102 exhibiting inconsistent behavior, creating a missing data bias that compromises national-level accuracy [10].

Furthermore, the system has structural blind spots that are particularly problematic for detailed research. SIPSN data collection is structured around formal, government-registered facilities (such as landfills and official waste banks) and largely fails to capture the massive contribution of Indonesia's informal waste sector. The critical work done by the middleman is also listed but has the potential to miss the unlisted middleman; this condition can be a problem for the data uncertainty for the calculation.

1. **RISKESDAS 2018**
2. **Understanding Riskesdas 2018: National Health Snapshot**

Riskesdas stands for *Riset Kesehatan Dasar*, which translates to Basic Health Research. It is a national-scale health survey conducted by the Health Research and Development Agency (Balitbangkes) of the Ministry of Health of the Republic of Indonesia. Its primary objective is to provide comprehensive data and information regarding the public's health status, disease risk factors, and access to healthcare services throughout Indonesia. The scale of the survey is massive, with data designed to be representative of the population not only at the national and provincial levels but also down to the district/city (*kabupaten/kota*) level. This makes the report critically important for local health development planning. This research is conducted periodically (e.g., 2007, 2013, 2018), allowing the government to monitor health trends and evaluate public health programs over time. The 2018 Riskesdas was particularly notable because it was integrated with the National Socioeconomic Survey (Susenas) from Statistics Indonesia (BPS), enabling richer analysis by linking health data directly with socioeconomic data [11].

1. **Methodology and Broad Scope**

The 2018 Riskesdas used a *cross-sectional* study design, capturing a snapshot of data at a single point in time. It surveyed hundreds of thousands of households, which is reflected in the large weighted sample size of 282,654 households. The data collected are far broader than just waste management; its main coverage includes the following:

- Nutritional status (e.g., stunting, child malnutrition)
- Communicable diseases (e.g., tuberculosis, diarrhea, malaria)
- Noncommunicable diseases (e.g., hypertension, diabetes, obesity, smoking)
- Maternal and Child Health (KIA)
- Access to and utilization of health services (e.g., national health insurance/JKN, immunization)

1. **The Household Waste Management Data**

In the context of waste management, Riskesdas 2018 provides crucial data on waste handling at the source (the household). It is critical to classify this as self-reported behavioral data, not perception data, as it captures what people say they do. The survey asks respondents to identify their primary disposal method, which is categorized as follows:

- Diangkut (Collected by a formal service)
- Dibakar (Burned)
- Ditanam (Buried)
- Dibuat kompos (Composted)
- Dibuang ke kali/selokan/laut (Dumped into rivers/drains/sea)
- Dibuang sembarangan (Dumped in an open space/illegally)

1. **Critical analysis: strengths and limitations of the data**

This dataset has immense value, but it must be used with a clear understanding of its strengths and limitations.

**Strengths**

- Statistical power: With a sample size of 282,654 households, its scale provides statistical power. This results in a very low confidence interval (often 1–5% at the municipal, provincial or national level), meaning that the estimates are highly precise.
- Cross-validation: These bottom-up household data can be used as powerful tools to crosscheck top-down official municipal data or figures from the National Waste Management Data System (SIPSN), which rely on reports from collection services and landfills.

**Limitations**

Despite its strengths, the data must be interpreted carefully owing to three primary limitations:

1. Social desirability bias: The data are entirely self-reported. A respondent may be embarrassed to admit to high-risk behavior, such as dumping waste directly into a river, which they know is socially "wrong" or polluting. Consequently, they might lie and claim a more "acceptable" practice, such as burning or burying, which skews the data.
2. Oversimplification (Primary Method): The survey forces respondents to choose only their *primary* method. This approach fails to capture the complex reality of mixed waste management, where a single household often uses multiple methods simultaneously (e.g., recycling valuables, composting organics, and burning plastics). The data flattens this complex behavior into a single, misleading category.
3. Lack of Causal Context (The "Why"): The most significant weakness is that the data provide no causal context; it tells us *what* households are doing but cannot explain *why*. The fact that households burn their waste is a critical finding, but it does not tell us if this is due to a service failure (unreliable/expensive collection), a cultural norm, a lack of risk perception (believing burning is "clean"), or an infrastructure barrier (living in a narrow alley). Without knowledge of "why," it is impossible to design an effective policy.

Nevertheless, despite these potential errors and biases, Riskesdas 2018 is the only comprehensive national dataset available at the regency/city level for household waste treatment. Meanwhile, data on waste treatment from non-domestic similar to Riskesdas sources is not yet available in Indonesia, therefore the data from SIPSN is used for non-domestic.

1. **Local Data**

The model's foundation was built upon national-level statistics from the SIPSN, but these data were systematically refined via a bottom-up triangulation approach. Beyond SIPSN, waste data is also available in the IKPLHD (Regional Environmental Quality Index) at provincial and regency/city levels, though not all IKPLHD reports are easily accessible via the websites of respective regional Environmental Agencies (DLH). Other potential sources include the Strategic Plans (Renstra) of related agencies and Regional Mid-Term Development Plans (RPJMD). However, the best data is typically found in the annually updated IKPLHD. These local data served two critical functions. First, it was used for gap filling where the SIPSN data were missing or outdated. Second, it enabled cross-validation to identify and correct discrepancies; for example, a Jakstrada policy document stating a measured collection rate of 45% was used to correct an aspirational 60% goal found in the national portal. This triangulation process proved essential for improving the accuracy of the formal collection rate and directly informed the model's data quality index (DQI).

1. **Sampling Data**

The final and most rigorous layer was the use of a new, primary ground truth dataset from a field sampling campaign conducted in 16 cities across Indonesia. This dataset was assumed to be the most accurate and was applied in two ways. First, for the 16 sampled cities, these primary data superseded all secondary sources. Our directly measured values were used for critical variables such as the plastic waste fraction per capita waste generation rates and household disposal behaviors; these cities served as high-confidence "anchor points" in the model. Second, and more powerfully, this dataset was employed for national-scale calibration. We also compared our field data to the SIPSN data for the same 16 cities to quantify systemic biases and create correction factors (e.g., SIPSN was found to systematically overestimate collection by 15%), which were then applied to similar regions nationwide to improve the accuracy of the entire model.

To assess the reliability of the secondary data used in the Material Flow Analysis (MFA), we conducted a comparative analysis between the official waste generation rates reported in the SIPSN database and primary field sampling data collected from 17 regencies and cities. The selection of these validation sites was explicitly restricted to regions with existing entries in the SIPSN database. This criteria was essential because the core formulation of this study relies on calibrating the uncertainty parameters of the national dataset. By selecting cities with established SIPSN values, we could quantitatively measure the deviation between reported values and ground-truth conditions. This deviation directly informs the Local Variation Coefficient (CV) used in our Monte Carlo uncertainty framework.

The analysis reveals that SIPSN data reliability is highly heterogeneous

- In Banyumas Regency, the SIPSN database reports a generation rate of only 0.29 kg/cap/day, whereas actual sampling reveals a rate of 0.57 kg/cap/day, a deviation of +96.8%. Similarly, Pontianak and Bangka Tengah are underestimated by approximately 50%.
- Conversely, Bukittinggi and Banda Aceh show negative deviations of approximately -50% to -55%, indicating that the national database significantly overestimates waste generation in these cities.
- Only a few locations, such as Manokwari, Kolaka, and Ternate, show high reliability with deviations under 10%.

| **No** | **Region** | **Sampling**  **(kg/cap/day)** | **SIPSN (kg/cap/day)** | | **Error (SIPSN-Actual)** | **Absolute Error** | **APE (%)** | **Relative Error** |
| --- | --- | --- | --- | --- | --- | --- | --- | --- |
| 1 | Banyuwangi | 0.38 | 0.46 | | 0.07 | 0.07 | 19.11 | 0.19 |
| 2 | Jembrana | 0.74 | 0.50 | | -0.24 | 0.24 | 32.65 | -0.33 |
| 3 | Cirebon | 0.36 | 0.60 | | 0.24 | 0.24 | 66.67 | 0.67 |
| 4 | Batu | 0.49 | 0.61 | | 0.11 | 0.11 | 23.22 | 0.23 |
| 5 | Bangka Tengah | 0.60 | 0.40 | | -0.20 | 0.20 | 33.50 | -0.33 |
| 6 | Batam | 0.58 | 0.89 | | 0.31 | 0.31 | 53.99 | 0.54 |
| 7 | Banda Aceh | 0.49 | 0.97 | | 0.48 | 0.48 | 97.36 | 0.97 |
| 8 | Bukittinggi | 0.45 | 1.01 | | 0.56 | 0.56 | 123.45 | 1.23 |
| 9 | Kotawaringin Timur | 0.65 | 0.53 | | -0.13 | 0.13 | 19.35 | -0.19 |
| 10 | Rembang | 0.43 | 0.38 | | -0.06 | 0.06 | 12.67 | -0.13 |
| 11 | Ternate | 0.37 | 0.35 | | -0.02 | 0.02 | 5.66 | -0.06 |
| 12 | Banyumasi | 0.57 | 0.29 | | -0.28 | 0.28 | 49.21 | -0.49 |
| 13 | Kolaka | 0.46 | 0.48 | | 0.02 | 0.02 | 3.46 | 0.03 |
| 14 | Pontianak | 0.89 | 0.59 | | -0.30 | 0.30 | 33.48 | -0.33 |
| 15 | Parepare | 0.44 | 0.48 | | 0.04 | 0.04 | 9.57 | 0.10 |
| 16 | Pasuruan | 0.52 | 0.71 | | 0.20 | 0.20 | 38.18 | 0.38 |
| 17 | Manokwari | 0.86 | 0.80 | | -0.06 | 0.06 | 7.08 | -0.07 |
|  | | | | | | | | |
| MAPE (%) | | | | 36.98 | | | | |
| Mean Bias Error (MBE) | | | | 0.04 | | | | |
| RMSE | | | | 0.25 | | | | |
| Std Dev of Error | | | | 0.25 | | | | |
| Mean of Actual | | | | 0.55 | | | | |
| CV of Reporting Error | | | | 0.46 | | | | |

These bidirectional discrepancies (ranging from -55% to +96%) empirically validate the necessity of the Multilayer Uncertainty Framework employed in this study. The fact that even official data points can deviate by nearly double (or half) the actual value justifies our application of wide uncertainty bounds (CV up to 50%) to ensure the final leakage model captures these inherent inconsistencies in the national reporting system.

1. **Data Harmonization, Correction and Quality Assurance**

To formally quantify the uncertainty in the plastic waste material flow analysis (MFA), a Monte Carlo simulation (MCS) with 10,000 iterations was employed. This methodology involves constructing a multilayer uncertainty framework by first deconstructing uncertainty into its fundamental components. A core feature of this approach is the separation of uncertainty into two distinct types: systematic (global) uncertainty and local (municipal) uncertainty. Systematic uncertainty accounts for a single, shared bias applied to all 514 municipalities simultaneously in each run (e.g., a systemic flaw in national data collection standards), whereas local uncertainty applies an independent random factor to each municipality to account for local errors and natural variability (e.g., a local data entry error or unique regional habits). All uncertain inputs were modeled via lognormal distributions to ensure that all the sampled values, such as waste generation, remained nonnegative.

Data from primary sampling is the most accurate compared to data derived from estimates or per capita waste generation multipliers. However, the data used to populate SIPSN or other waste data documents predominantly relies on assumptions or multipliers. This is because primary sampling requires significantly more funding than other data analysis methods, leading to a higher potential for bias or error compared to primary data. Previous state that obtaining accurate field data on waste statistics—including quantity, composition, characteristics, and recycling potential—is recognized by many experts as a major challenge in developing countries [12]. Nevertheless, the only comprehensive source for waste data in Indonesia at present is SIPSN.

Data on waste generation is a primary dataset in this study, but other data is crucial for understanding waste treatment and management from its source to final processing at a TPA or TPST. The data in SIPSN also covers both formal and informal waste facilities, including TPS3R, waste banks, PDU, composting, Waste-to-Energy (WTE) facilities, TPST, and TPA. However, some of this data is not updated annually and is sometimes duplicated from the previous year.

Furthermore, based on analysis and observation of the data from 2018 to 2023, several anomalous values were identified for waste generation or the volume of waste managed at facilities. For example, some facilities report waste intake volumes that are significantly larger than their processing capacity, or the total waste entering TPSTs/TPAs exceeds the total waste generation for that entire regency/city. This is a critical issue to address in Material Flow Analysis (MFA). Normalization and data cleaning are required to obtain more accurate and reasonable data.

The input parameters were characterized by combining baseline data with these uncertainty factors. Both the baseline waste generation and the waste management fractions (e.g., collected, open burning, river disposal) were assigned a systematic (global) CV of 0.10 (10%). This represented moderate, system-wide bias from the use of national-level standards (SNI) and perception-based national surveys (RISKESDAS). For local (municipal) uncertainty, the waste management fractions were assigned a CV of 0.20 (20%). This higher value was justified because the underlying RISKESDAS data are old, perception-based, and not locally specific, thus carrying high uncertainty when applied at the municipal level. In contrast, the local CV for waste generation was variable and tied directly to a data quality index (DQI). This CV ranged from 0.10 (10%) for the highest-quality data (e.g., primary field sampling) to 0.50 (50%) for the lowest-quality data (e.g., imputed data from very old sources). In every iteration, the resulting waste management fractions were normalized to ensure that they summed to 100%, thus maintaining mass balance.

**Table S2.** Uncertainty number of the model

| **Parameter** | **Uncertainty Type** | **CV** | **Justification** |
| --- | --- | --- | --- |
| Waste Generation | Systematic (Global) | 0.10 (10%) | Models a moderate, system-wide bias in all generation data. This accounts for potential shared flaws in the national standards for waste sampling or their inconsistent application across the country. |
| Waste Management Fractions  *(Collected, Burned, Direct Disposal, etc.)* | Systematic (Global) | 0.10 (10%) | Models a moderate, system-wide bias in the behavioral data. This is directly linked to using a single national survey (like RISKESDAS) and SIPSN. Any flaw in the survey's "perception-based" questions would be shared by all 514 regions. |
| Waste Management Fractions | Local (Municipal) | 0.20 (20%) | This reflects the high local uncertainty of using old, national, perception-based data. Even if the RISKESDAS national average has a 95% CI, applying it to a *specific* regency in the *current* year is highly uncertain. This 20% CV is a justified "penalty" for data that is (a) old, (b) perception-based, and (c) not locally specific. |
| Waste Generation | Local (Municipal) | Variable | This is the Data Quality Index (DQI) in action. The local uncertainty in waste generation is *not* one-size-fits-all; it is tied directly to the *source* of the data for that specific municipality. |
|  | *DQI 5 (Best* | *0.10 (10%)* | Best Quality: Represents the 17-city primary field sampling. This data is based on direct measurement, so it is assigned the lowest uncertainty. |
|  | *DQI 4 (High)* | *0.20 (20%)* | High Quality: Represents recent (e.g., 2022) SIPSN data, likely cross-validated with local Environmental Agency reports |
|  | *DQI 3 (Medium)* | *0.30 (30%)* | Medium Quality: Represents older SIPSN data (e.g., 2021). Confidence decreases as the data ages and no longer reflects the most current conditions. |
|  | *DQI 2 (Low)* | *0.40 (40%)* | Low Quality: Represents very old SIPSN data (e.g., 2019-2020). This data is considered unreliable as it predates major economic/social shifts. |
|  | *DQI 1 (Worst)* | *0.50 (50%)* | Worst Quality: Represents missing data that had to be imputed (i.e., estimated based on neighbors or old values). It is assigned the highest uncertainty. |

This simulation process generated a full probability distribution for all key outputs. The final results were reported not as single point estimates but as a median (50th percentile) value accompanied by a 95% confidence interval (CI), which captures the full range of plausible outcomes.

1. **Data Comparative Benchmarking, Callibration and Validation**

To ensure the robustness and predictive accuracy of the proposed material flow model, this study employs a verification framework consisting of three distinct analytical phases: comparative benchmarking, input calibration, and validation. First, a data comparison is conducted to juxtapose the model’s outputs against the Sistem Informasi Pengelolaan Sampah Nasional (SIPSN) baseline. This step serves to identify discrepancies between the model's granular, field-based estimations and the generalized coefficients used in national reporting, specifically highlighting deviations in heterogeneous zones such as industrial-metropolitan districts. Second, data calibration is performed to verify the reliability of the primary field sampling data used as model input. By cross-referencing sampling results with independent values from previous studies, the study ensures that input parameters reflect established local waste generation trends rather than transient anomalies. Finally, data validation is executed to confirm the model's fidelity. By demonstrating that the model’s outputs, derived from these calibrated inputs, align more closely with historical empirical evidence than the SIPSN standards, the model is validated as a reliable tool for localized waste management planning.

1. Comparative Benchmarking (Model based on Sampling vs SIPSN)

The initial phase evaluates the divergence between the model's stochastic projections (Median, P5, P95) and the deterministic values provided based on SIPSN calculation. The figure S.1 is shown the comparison between the waste generation rate and plastic waste fraction between SIPSN and field sampling.


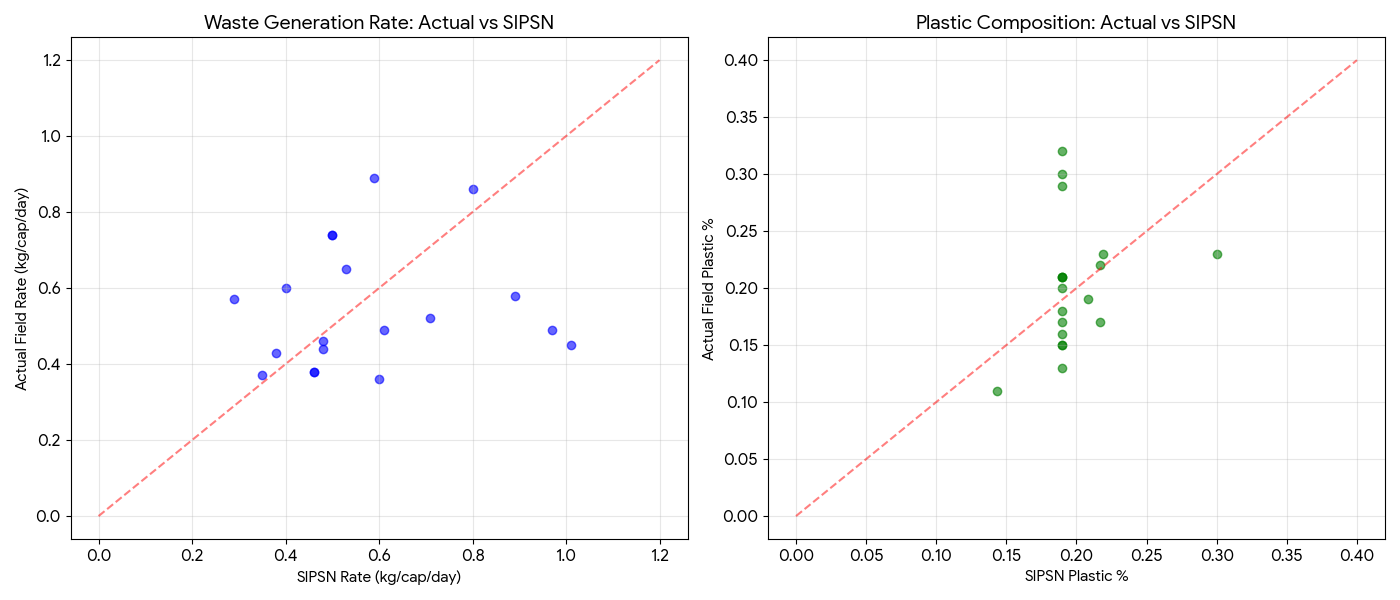


Figure S.1 Waste Generation Rate and Plastic Composition Rate

Based on the comparative analysis of specific subdistricts, the reliability of the proposed model is substantiated by its ability to resolve the systematic inaccuracies inherent in the SIPSN baseline. The data reveals that the standardized SIPSN methodology frequently produces estimates that are statistically improbable for specific locations, whereas the model captures the granular reality of waste generation. The comparison between Model and SIPSN based calculation is shown in Figure S.2.


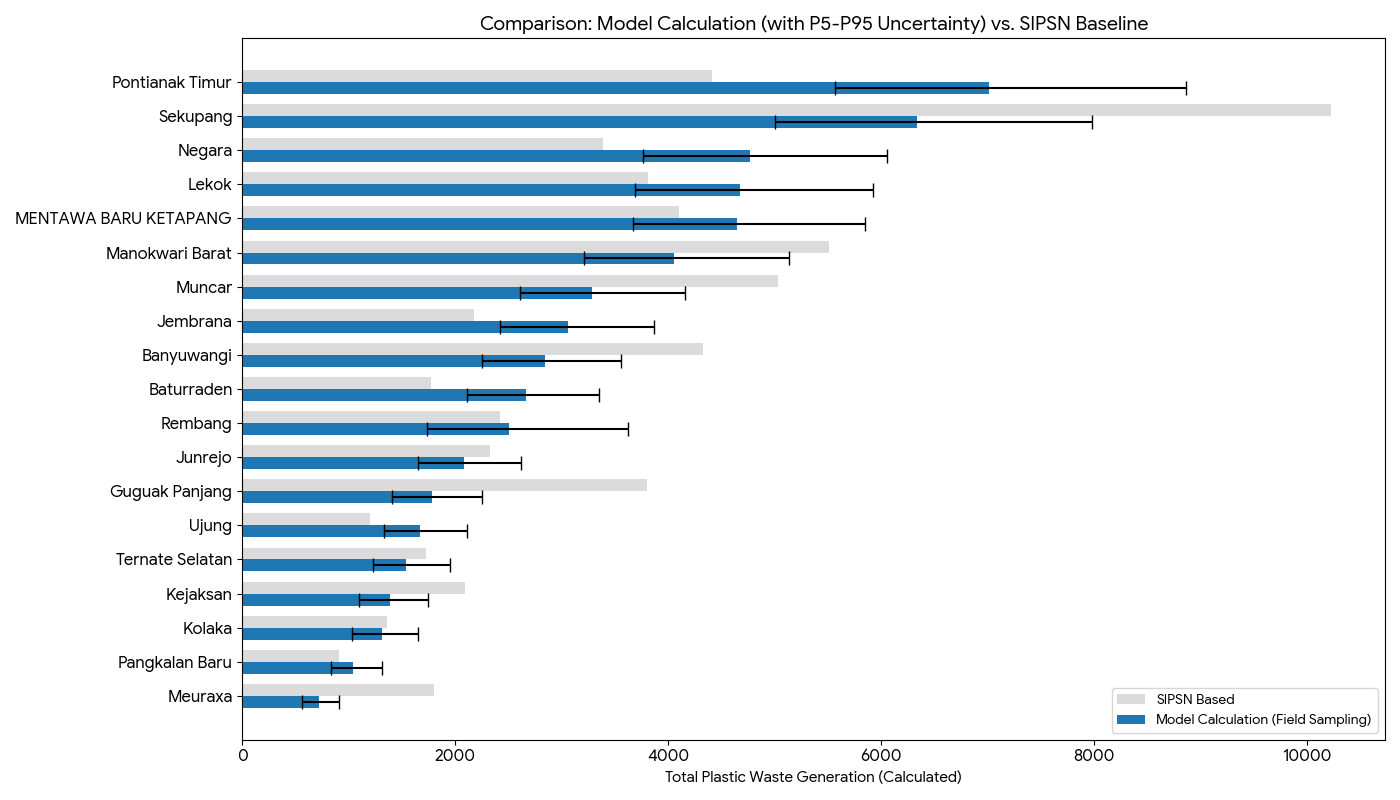


Figure S.2 Comparison of Model Result vs SIPSN calculation

Meanwhile, if we compare the uncalibrated model vs SIPSN estimates. This model estimates that the total plastic waste generation is 8.02% lower than what the SIPSN estimation predicts. This suggests a slight but significant tendency for the national standard (SIPSN) to overestimate the total waste burden across the sampled (sub-district), largely driven by the over-predictions in major areas like Sekupang and Banyuwangi.

Meanwhile based on the specific location in each sub district (Table S.2), a primary indicator of the model's reliability is its capacity to correct significant overestimations in waste volume, particularly in urban and metro-industrial zones. In districts such as Sekupang and Banyuwangi, the SIPSN estimates deviate positively by +61.2% and +52.4% respectively. In both instances, the SIPSN values fall outside the model’s upper confidence limit (>P95), classifying them as statistical outliers. This suggests that the national standard relies on generalized coefficients that conflate potential capacity with actual generation, leading to inflated projections.

Table S.2. Comparison of Specific Location

| **Kecamatan**  **(Sub-District)** | **Typology** | **SIPSN Estimate (kg/day)** | **Model Median (kg/day)** | **Deviation (%)** | **Statistical Status** |
| --- | --- | --- | --- | --- | --- |
| Sekupang | Urban (Metro) | 10,223.47 | 6,339.75 | +61.2% | Outlier (>P95) |
| Pontianak Timur | Urban | 4,414.77 | 7,015.91 | -37.1% | Outlier (<P5) |
| Banyuwangi | Urban | 4,327.30 | 2,838.77 | +52.4% | Outlier (>P95) |
| Lekok | Rural | 3,813.61 | 4,678.65 | -18.5% | Within Range |
| Muncar | Rural | 5,028.56 | 3,288.68 | +52.9% | Outlier (>P95) |
| Negara | Semi Urban | 3,387.75 | 4,769.01 | -28.9% | Outlier (<P5) |
| Manokwari Barat | Urban | 5,509.00 | 4,050.97 | +36.0% | Within Range |

Conversely, the model demonstrates sensitivity in detecting waste generation that are underestimated by the national standard. In Pontianak Timur (Urban) and Negara (Semi-Urban), the SIPSN estimates are significantly lower than the model’s median calculation, with deviations of -37.1% and -28.9% respectively. In these cases, the SIPSN values fall below the model's lower statistical bound (<P5), indicating a failure to account for local consumption intensity or high-density behaviors. The model’s ability to capture this "hidden" waste load is critical for preventing operational failures, such as the under-sizing of collection fleets or disposal cells, which would occur if planning relied solely on the SIPSN baseline.

1. Input Calibration via Historical Data

Recognizing that the predictive fidelity of any material flow model is intrinsically linked to the quality of its primary inputs, this study implements a rigorous calibration framework benchmarks field sampling data against historical evidence from well-documented subdistricts. To ensure analytical precision, the validation process was stratified across two critical dimensions: spatial typology (Urban vs. Rural) and data scope (Household Solid Waste vs. Total Solid Waste). This distinction is paramount because the National Waste Management Information System (SIPSN) typically reports a Total Solid Waste Equivalent—a metric often derived from generic regional averages to impute data gaps. Consequently, a direct comparison between specific household sampling and SIPSN’s aggregate values can lead to false equivalencies.

To resolve this disparity, we compiled previous studies to benchmark waste generation and plastic composition across several subdistricts (Table S.3). However, a significant compatibility challenge emerged, many historical studies provide only household-based sampling, rendering them utilizing a narrower system boundary than the total waste generation scope employed in this study. Therefore, a calibration factor was calculated to align these datasets. To demonstrate the necessity of this adjustment, an initial assessment was conducted using the raw, uncalibrated model. The resulting baseline deviation between the uncalibrated model and the sampling studies is presented in Figure S.3.


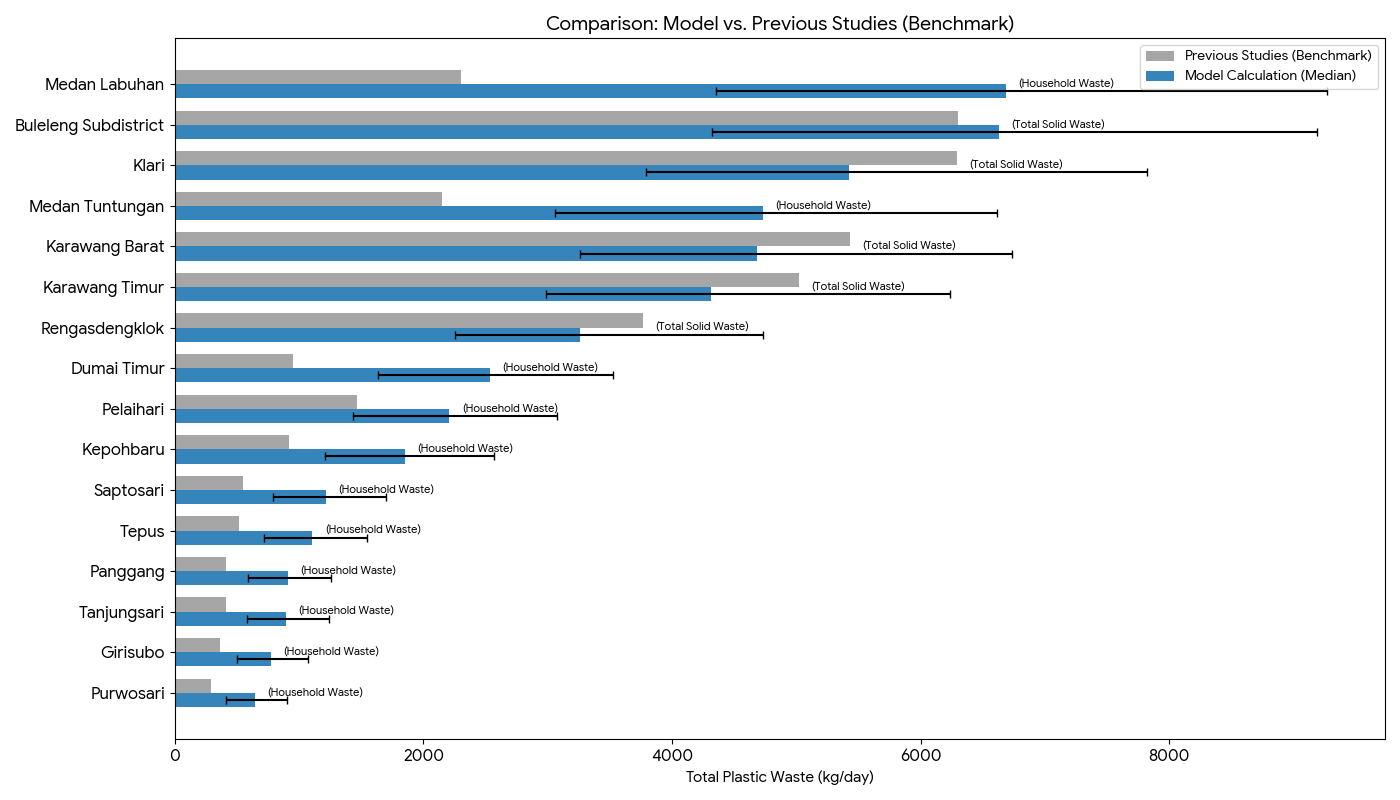


Figure S.3 Comparison Between Uncalibrated Model vs Previous Studies

Table S.3 Comparison Between Prvious Studies and Model

| **Kecamatan** | **Actual (kg/cap/**  **day)** | **Plastic (%)**  **(Prev. Studies)** | **Scope**  **(Prev. Studies)** | **SIPSN (kg/cap/**  **day)** | **Plastic (%)**  **(SIPSN)** | **Pop.** | **Typical Sub-district** | **Previous Studies** | **SIPSN Based** | **Model Result Median** | **P5 (5%)** | **P95 (95%)** | **Source** |
| --- | --- | --- | --- | --- | --- | --- | --- | --- | --- | --- | --- | --- | --- |
| Buleleng Subdistrict | 0.74 | 15% | TSW* | 0.62 | 19% | 155,025 | Urban | 6297.59 | 6672.63 | 6627.56 | 4319.21 | 9187.69 | [13] |
| Dumai Timur | 0.314 | 12% | HHW** | 0.504 | 19% | 72,956 | Urban | 952.85 | 2552.67 | 2533.02 | 1636.28 | 3526.97 | [14] |
| Purwosari | 0.40 | 9% | HHW | 0.44 | 19% | 21,267 | Rural | 289.41 | 649.62 | 644.21 | 416.31 | 902.28 | [15] |
| Panggang | 0.40 | 9% | HHW | 0.44 | 19% | 29,942 | Rural | 410.16 | 914.61 | 909.43 | 593.15 | 1257.60 | [15] |
| Saptosari | 0.40 | 9% | HHW | 0.44 | 19% | 40,251 | Rural | 551.51 | 1229.51 | 1214.30 | 789.99 | 1702.00 | [15] |
| Tanjungsari | 0.40 | 9% | HHW | 0.44 | 19% | 29,524 | Rural | 409.75 | 901.84 | 896.82 | 585.13 | 1240.64 | [15] |
| Tepus | 0.40 | 9% | HHW | 0.44 | 19% | 36,639 | Rural | 514.81 | 1119.18 | 1108.10 | 719.75 | 1546.46 | [15] |
| Girisubo | 0.40 | 9% | HHW | 0.44 | 19% | 25,588 | Rural | 361.51 | 781.61 | 771.39 | 498.22 | 1069.65 | [15] |
| Kepohbaru | 0.25 | 15% | HHW | 0.4 | 19% | 67,420 | Rural | 922.81 | 1872.20 | 1854.45 | 1209.64 | 2564.78 | [16] |
| Pelaihari | 0.36 | 14% | HHW | 0.401 | 19% | 79,911 | Rural | 1470.04 | 2224.61 | 2209.60 | 1436.10 | 3077.37 | [17] |
| Karawang Barat | 0.42 | 21% | TSW | 0.4 | 19% | 168,734 | Urban | 5432.05 | 4685.61 | 4682.87 | 3259.73 | 6733.31 | [18] |
| Karawang Timur | 0.42 | 21% | TSW | 0.4 | 19% | 156,029 | Urban | 5023.04 | 4332.80 | 4314.76 | 2986.57 | 6238.45 | [18] |
| Klari | 0.42 | 21% | TSW | 0.4 | 19% | 195,489 | Urban | 6293.38 | 5428.57 | 5427.91 | 3794.40 | 7818.16 | [18] |
| Rengasdengklok | 0.42 | 21% | TSW | 0.4 | 19% | 117,087 | Urban | 3769.38 | 3251.41 | 3257.77 | 2255.33 | 4729.83 | [18] |

*TSW = Total Solid Waste

**HHW = Household Solid Waste

Benchmarking the model against previous studies that share an identical system boundary, specifically those reporting Total Solid Waste Equivalent, the predictive accuracy is exceptionally high. In regions such as Buleleng, Karawang, and Rengasdengklok, the Mean Absolute Percentage Error is recorded at 10%. This strong alignment serves as the primary validation of the core algorithm, demonstrating that when historical data captures the full spectrum of waste generation, the model correctly predicts the total waste load for a subdistrict with high precision. This confirms that the coefficients for total generation within the model are calibrated correctly against comprehensive field data.

Conversely, a significant divergence is observed when comparing the model against studies with a limited scope, specifically those measuring only Household Waste. In instances like Dumai Timur, and Purwosari, the high Mean Absolute Percentage Error of 73% does not indicate a model failure but rather a structural mismatch in sampling scope. Because these previous studies systematically excluded non-domestic sources such as commercial markets and public facilities, they inherently underestimate the total regional waste generation. The model, which accounts for the combined load of household and non-domestic sources, naturally yields higher values. Therefore, this statistical deviation effectively quantifies the non-domestic fraction that was previously unmeasured. Far from being a flaw, this deviation validates that the model is successfully capturing the significant volume of non-household waste that restricted-scope studies fail to report, thereby providing a more realistic and comprehensive profile of total plastic waste leakage. The MAPE analysis is shown in Table S.4.

To address the significant divergence observed in household-only studies, a secondary adjustment stage was introduced. This stage applies a Non-Domestic Correction Factor to the restricted-scope datasets, effectively match the previous studies Total Solid Waste Equivalent. The initial benchmarking revealed that historical studies limited to Household Waste consistently underestimated the regional waste load compared to the model, which captures the full system. To resolve this structural scope mismatch, it is imperative to quantify the missing non-domestic fraction—waste generated by markets, commercial centers, offices, and public facilities—and incorporate it into the household-only datasets. This correction ensures that all historical benchmarks are converted into a Total Solid Waste Equivalent before being compared to the model.

To establish a statistically valid correction factor, a supplementary dataset was analyzed comprising 10 municipalities where both Household and Total Waste Generation rates were explicitly measured. The previous studies to obtain this factor is shown is shown in Table S.4.

Table S.4. Comparison between Household and Total Waste Generation

| **No** | **City/Regency** | **Typology** | **HH Generation (kg/cap/day)** | **Total PW**  **(domestic and non-domestic) (kg/cap/day)** | **Derived Non-Domestic Ratio** | **Source** |
| --- | --- | --- | --- | --- | --- | --- |
| 1 | Denpasar | Urban | 0.32 | 0.81 | 0.60 | [19] |
| 2 | Badung | Urban | 0.40 | 0.74 | 0.46 | [19] |
| 3 | Jatinegara | Urban | 0.26 | 0.37 | 0.30 | [20] |
| 4 | Karawang | Urban | 0.42 | 0.56 | 0.25 | [18] |
| **Urban average** | | | | | **0.45** |  |
| 5 | Jembrana | Rural | 0.31 | 0.46 | 0.33 | [19] |
| 6 | Karangasem | Rural | 0.30 | 0.38 | 0.21 | [19] |
| 7 | Bangli | Rural | 0.25 | 0.37 | 0.32 | [19] |
| 8 | Buleleng | Rural | 0.39 | 0.47 | 0.17 | [19] |
| 9 | Gianyar | Rural | 0.28 | 0.36 | 0.22 | [19] |
| 10 | Tabanan | Rural | 0.32 | 0.40 | 0.20 | [19] |
| **Rural average** | | | | | **0.24** |  |

This dual-measurement dataset allows for the isolation of the non-domestic contribution. The analysis stratified the data by typology, revealing a distinct split in waste composition. Urban centers, such as Denpasar and Badung, exhibit a significantly higher non-domestic fraction due to the density of commercial activity, tourism, and institutional presence. Consequently, the analysis derived an Urban Non-Domestic Ratio of 0.45, indicating that 45% of total waste originates from non-household sources. Conversely, rural areas like Banyuwangi and Tabanan showed a much lower dependency on non-domestic sources, yielding a Rural Non-Domestic Ratio of 0.25. This aligns with rural characteristics where waste is predominantly residential and agricultural, with fewer commercial hubs. The calibrated number for rural type areas is 0.8 and 1 for urban and semi urban.

Following calibration, the overall MAPE decreased from 46.79% to 30.69%, representing a 16.1 percentage-point reduction and a 34% relative improvement in predictive accuracy. Rural–HHW improved substantially from 73.33% to 40.10%, demonstrating the effectiveness of the correction in reducing structural bias, although variability remains comparatively higher in rural contexts. Semi-Urban–TSW remained stable at 13.57%, confirming structural robustness, while Urban–TSW showed strong and consistent performance with a calibrated MAPE of 9.64%. Urban–HHW, however, exhibited persistent overestimation at 76.11%, indicating typology-specific uncertainty rather than systemic model failure. Importantly, all calibrated outputs fall within the stochastic confidence bounds (P5–P95), and additional validation metrics confirm improvement. The overall comparison of MAPE between Previous Studies with Uncalibrated and Calibrated Model is shown in Table S.5.

Table S.5 The MAPE between Previous Studies with Uncalibrated and Calibrated Model

| **Category** | **MAPE Uncal (%)** | **MAPE Cal (%)** | **Interpretation** |
| --- | --- | --- | --- |
| Overall Model | 46.79 | 30.69 | Calibration improved accuracy substantially |
| Rural – HHW | 73.33 | 40.10 | Significant improvement but still high variability |
| Semi Urban – TSW | 13.57 | 13.57 | Structurally stable, no calibration change |
| Urban – HHW | 74.10 | 76.11 | Persistent overestimation |
| Urban – TSW | 10 | 10 | Strong and consistent performance |

The calibration process also materially influenced national plastic waste flow magnitudes. Total generated plastic waste decreased from 10,664,251 ± 1,605,539 tonnes per year in the uncalibrated scenario to 9,219,831 ± 1,410,521 tonnes per year after calibration. Correspondingly, collected waste declined to 3,776,543 ± 580,790 tonnes per year, openly burned waste to 4,159,740 ± 638,269 tonnes per year, disposal to rivers and drains to 643,101 ± 98,350 tonnes per year, and dumping to open land to 521,526 ± 80,517 tonnes per year. The overall comparison of result between uncalibrated to calibrated model is shown in Table S.6.

Table S.6. Overall Comparison Uncalibrated With Calibrated Model

| **Flow Category**  **(Plastic Waste)** | **Plastic Waste Flow**  **(tonnes/year)**  **Uncalibrated** | **Plastic Waste Flow**  **(tonnes/year)**  **Calibrated** |
| --- | --- | --- |
| Total Generated | 10,664,251 ± 1,605,539 | 9,219,831 ± 1,410,521 |
| Collected | 4,099,385 ± 619,329 | 3,776,543 ± 580,790 |
| Buried | 147,575 ± 22,772 | 121,032 ± 18,660 |
| Openly Burned | 5,006,141 ± 754,027 | 4,159,740 ± 638,269 |
| Disposed Directly to Rivers/Drains | 777,730 ± 116,842 | 643,101 ± 98,350 |
| Dumped Directly to Terrestrial (Open Land) | 633,693 ± 95,703 | 521,526 ± 80,517 |

These reductions indicate that uncalibrated coefficients may overstate leakage pathways and environmental burdens. Overall, the integrated benchmarking, calibration, and validation framework confirms that the proposed material flow model achieves moderate overall predictive accuracy (MAPE 30.69%) and strong structural performance in total solid waste contexts, while transparently identifying typology-specific sensitivities. This comprehensive verification approach substantiates the model as a statistically reliable and policy-relevant tool for subdistrict-level plastic waste planning and leakage assessment.

1. **Output Data from Monte Carlo Simulation**

📊 NATIONAL AGGREGATED RESULTS (Ton per Year)

TOTAL GENERATION | Median: 9,205,297 | 5%: 7,816,619 | 95%: 10,859,487

Managed | Median: 3,771,502 | 5%: 3,199,445 | 95%: 4,443,948

Buried | Median: 121,079 | 5%: 102,311 | 95%: 142,776

Open_Burning | Median: 4,153,970 | 5%: 3,527,559 | 95%: 4,895,577

Disposal_River | Median: 641,841 | 5%: 544,162 | 95%: 756,852

Disposal_Land | Median: 520,535 | 5%: 442,472 | 95%: 613,415

TYPOLOGY BREAKDOWN (Ton per Year)

>>> RURAL

TOTAL GENERATION | Median: 5,841,870 | 5%: 4,958,572 | 95%: 6,882,054

Managed | Median: 1,314,706 | 5%: 1,115,844 | 95%: 1,548,178

Buried | Median: 105,461 | 5%: 89,344 | 95%: 124,405

Open_Burning | Median: 3,429,743 | 5%: 2,911,856 | 95%: 4,041,152

Disposal_River | Median: 544,807 | 5%: 461,429 | 95%: 642,587

Disposal_Land | Median: 447,681 | 5%: 380,439 | 95%: 527,272

>>> URBAN

TOTAL GENERATION | Median: 3,300,435 | 5%: 2,800,412 | 95%: 3,894,290

Managed | Median: 2,432,264 | 5%: 2,061,989 | 95%: 2,871,706

Buried | Median: 14,796 | 5%: 12,007 | 95%: 18,297

Open_Burning | Median: 691,682 | 5%: 586,228 | 95%: 818,410

Disposal_River | Median: 93,364 | 5%: 78,519 | 95%: 111,805

Disposal_Land | Median: 68,975 | 5%: 57,827 | 95%: 82,429

>>> SEMI URBAN

TOTAL GENERATION | Median: 64,497 | 5%: 54,066 | 95%: 77,214

Managed | Median: 24,046 | 5%: 19,712 | 95%: 29,422

Buried | Median: 734 | 5%: 455 | 95%: 1,155

Open_Burning | Median: 31,880 | 5%: 26,396 | 95%: 38,427

Disposal_River | Median: 3,717 | 5%: 2,934 | 95%: 4,746

Disposal_Land | Median: 4,078 | 5%: 3,229 | 95%: 5,134

**References**

1. Cottom, J. W., Cook, E. & Velis, C. A. A local-to-global emissions inventory of macroplastic pollution. *Nature* **633**, 101–108 (2024).

2. Chen, Q. & Fei, X. Effective reduction of land-to-ocean plastic leakage in Thailand from 2000 to 2019 and implications for low- and middle-income countries. *Resources, Conservation and Recycling* **198**, 107204 (2023).

3. Jambeck, J. R. *et al.* Plastic waste inputs from land into the ocean. *Science* **347**, 768–771 (2015).

4. Lebreton, L. C. M. *et al.* River plastic emissions to the world’s oceans. *Nature Communications* **8**, (2017).

5. Meijer, L. J. J., Emmerik, T. van, Ent, R. van der, Schmidt, C. & Lebreton, L. More than 1000 rivers account for 80% of global riverine plastic emissions into the ocean. *Science Advances* **7**, (2021).

6. World Bank. *Plastic Waste Discharges From Rivers and Coatlines in Indonesia*. (Washington DC, 2021).

7. Liu, Y., Akashi, F. & Taniguchi, M. Quantile Method for Time Series. in *Empirical Likelihood and Quantile Methods for Time Series: Efficiency, Robustness, Optimality, and Prediction* (eds Liu, Y., Akashi, F. & Taniguchi, M.) 59–86 (Springer, Singapore, 2018). doi:10.1007/978-981-10-0152-9_3.

8. Smith, M. J. D., Goodchild, M. F. & Longley, P. A. *Geospatial Analysis: A Comprehensive Guide to Principles, Techniques and Software Tools*. (Winchelsea Press, 2018).

9. KLHK. SIPSN - Sistem Informasi Pengelolaan Sampah Nasional. https://sipsn.menlhk.go.id/sipsn/public/data/timbulan.

10. Purba, S. U. The difference in waste generation reporting compliance of districts/cities before and after the issuance of the Ministry of Environment and Forestry Regulation Number 6 of 2022. *TROPHICO* **5**, 96–101 (2025).

11. Kemenkes RI. Riset Kesehatan Dasar (Riskesdas) (2018). (2018).

12. Guerrero, L. A., Maas, G. & Hogland, W. Solid waste management challenges for cities in developing countries. *Waste Management* **33**, 220–232 (2013).

13. Widyarsana, I. M. W. & Rahayu, M. A. I. Solid waste management planning for sub-district scale: a case study in Buleleng sub-district, Buleleng district, Bali province, Indonesia. *J. Mater. Cycles Waste Manage.* **23**, 2051–2064 (2021).

14. Hidayat, F., Martono, D. N. & Hamzah, U. S. Quantification and characterization of household waste in Dumai Timur District, Dumai City, Riau, Indonesia as a measure towards circular economy. *IOP Conf. Ser.: Earth Environ. Sci.* **1094**, 012001 (2022).

15. Masjhoer, J. M., Syafrudin, S. & Maryono, M. Rural Waste Reduction Potential in The South of Gunungkidul Regency. *E3S Web Conf.* **448**, 03056 (2023).

16. Hidayah, N., Meidiana, C., Firdausiyah, N., Ghosh, R. K. & Amalia, R. STATE OF THE RURAL COMMUNITY ON THE LADDER OF PUBLIC PARTICIPATION: CASE STUDY WASTE MANAGEMENT PROGRAM IN MOJOSARI VILLAGE, BOJONEGORO REGENCY. *Journal of Environmental Engineering and Sustainable Technology* **11**, 93–102 (2024).

17. Yustina, Y., Suyanto, S., Mahyudin, R. P. & Sofia, L. A. ANALISIS TIMBULAN, KOMPOSISI DAN ARAHAN PENGELOLAAN SAMPAH DI KECAMATAN PELAIHARI KABUPATEN TANAH LAUT. *ES* **17**, 186–190 (2021).

18. Salman, N., Iskandar, A., Noviyanti, E. & Mellyanawaty, M. Waste Generation and Composition in Karawang Regency. *Journal of Community Based Environmental Engineering and Management* **8**, 67–74 (2024).

19. Widyarsana, I. M. W., Damanhuri, E. & Agustina, E. Municipal solid waste material flow in Bali Province, Indonesia. *J. Mater. Cycles Waste Manage.* **22**, 405–415 (2020).

20. Kuntaryo, A. M., Purwaningrum, P., Tazkiaturrizki, Minarti, A. & Ashardiono, F. THE RECYCLING POTENTIAL OF SOLID WASTE IN JATINEGARA SUB-DISTRICT, EAST JAKARTA. *INDONESIAN JOURNAL OF URBAN AND ENVIRONMENTAL TECHNOLOGY* 100–113 (2023) doi:10.25105/urbanenvirotech.v6i1.14793.
